# A Secure IIoT Environment That Integrates AI-Driven Real-Time Short-Term Active and Reactive Load Forecasting with Anomaly Detection: A Real-World Application

**DOI:** 10.3390/s24237440

**Published:** 2024-11-21

**Authors:** Md. Ibne Joha, Md Minhazur Rahman, Md Shahriar Nazim, Yeong Min Jang

**Affiliations:** Department of Electronics Engineering, Kookmin University, Seoul 02707, Republic of Korea; ibne.joha17@gmail.com (M.I.J.); minhaz.eee.97@gmail.com (M.M.R.); shahriarnazim45@gmail.com (M.S.N.)

**Keywords:** Industrial Internet of Things (IIoT), load forecasting, anomaly detection, energy management system, security, edge server, cloud server

## Abstract

The Industrial Internet of Things (IIoT) revolutionizes both industrial and residential operations by integrating AI (artificial intelligence)-driven analytics with real-time monitoring, optimizing energy usage, and significantly enhancing energy efficiency. This study proposes a secure IIoT framework that simultaneously predicts both active and reactive loads while also incorporating anomaly detection. The system is optimized for real-time deployment on an edge server, such as a single-board computer (SBC), as well as on a cloud or centralized server. It ensures secure and reliable industrial operations by integrating smart data acquisition systems with real-time monitoring, control, and protective measures. We propose a Temporal Convolutional Networks-Gated Recurrent Unit-Attention (TCN-GRU-Attention) model to predict both active and reactive loads, which demonstrates superior performance compared to other conventional models. The performance metrics for active load forecasting are 0.0183 Mean Squared Error (MSE), 0.1022 Mean Absolute Error (MAE), and 0.1354 Root Mean Squared Error (RMSE), while for reactive load forecasting, the metrics are 0.0202 (MSE), 0.1077 (MAE), and 0.1422 (RMSE). Furthermore, we introduce an optimized Isolation Forest model for anomaly detection that considers the transient conditions of appliances when identifying irregular behavior. The model demonstrates very promising performance, with the average performance metrics for all appliances using this Isolation Forest model being 95% for Precision, 98% for Recall, 96% for F1 Score, and nearly 100% for Accuracy. To secure the entire system, Transport Layer Security (TLS) and Secure Sockets Layer (SSL) security protocols are employed, along with hash-encoded encrypted credentials for enhanced protection.

## 1. Introduction

The Industrial Internet of Things (IIoT) refers to the integration of various sensors, devices, and machines connected to the Internet, enabling real-time monitoring, data acquisition, processing, and communication, which enhances efficiency, automation, and intelligent decision-making across diverse sectors such as automotive, healthcare, agriculture, construction, and energy management [1]. Industry 4.0 and the Industrial Internet of Things (IIoT) are interconnected frameworks that make use of cyber–physical systems and advanced data analytics to enhance industrial operations through autonomous decision-making and real-time optimization [2]. However, the recent evolution of the IIoT integrates advanced technologies such as artificial intelligence (AI), edge and cloud computing, machine learning, digital twins, federated learning, and blockchain to enhance industrial processes through interconnected smart devices, real-time analytics, virtual simulations, collaborative learning, and secure data management [3]. Besides emphasizing collaboration between human creativity and intelligent machines, Industry 5.0 aims to achieve efficiency and reduce human intervention, marking a significant advancement from previous industrial revolutions [4]. This evolution integrates state-of-the-art technologies such as AI, edge computing, digital twins, and collaborative robotics within a framework that prioritizes sustainability and environmental responsibility, setting a new standard for industrial innovation and societal impact [5,6]. Additionally, the increasing role of IoT in smart homes and industrial automation emphasizes the need for communication technologies that prioritize low latency and low energy consumption to ensure efficient performance [7].

In recent years, the impact of climate change has underscored the urgency of shifting towards sustainable energy sources and implementing smart solutions to manage global energy consumption effectively. Climate change is projected to significantly alter energy consumption patterns globally by mid-century, with regions experiencing varied impacts on demand across residential, commercial, and industrial sectors [8]. This increase in energy usage not only exacerbates carbon emissions but also underscores the urgent need for adaptive strategies and sustainable energy policies to mitigate climate impacts on energy consumption [9,10]. As a result of concern over energy consumption, implementing advanced home energy management systems (HEMS), such as AI-HEMS, represents a crucial strategy to effectively reduce household energy consumption, thereby potentially mitigating global warming by minimizing carbon emissions associated with excessive energy use [11]. An energy management system (EMS) or home automation system (HAS) typically follows a centralized, hierarchical structure where the communication layer plays a crucial role in ensuring its efficient operation [12]. Furthermore, integrating IIoT technologies into energy management systems enhances operational efficiency and resilience, which are essential for mitigating environmental impacts through optimized energy consumption strategies [13,14]. However, ensuring secure communication between IoT devices poses a significant challenge in today’s landscape, further exacerbating security concerns [15,16,17]. Robust security measures are imperative to safeguard data integrity, protect against cyber threats, and preserve privacy, thereby ensuring optimal network performance [18,19,20,21].

AI facilitates real-time data analysis, intelligent decision-making, and predictive analytics in addition to enhancing the security of the IIoT ecosystem against substantial cyber threats [22,23]. Furthermore, artificial intelligence (AI) and machine learning algorithms play a crucial role in optimizing resource allocation, ensuring grid stability and reliability, and enhancing energy usage planning [18,24]. These technologies enable accurate short-term load forecasting (STLF) in smart grid systems, facilitating efficient management of electricity demand and supply [25,26]. By analyzing complex data from smart meters and other sources, AI-driven approaches can predict load patterns with higher precision, thereby supporting decisions on storage device requirements and improving economic outcomes for both consumers and providers [27,28]. On the other hand, AI-based anomaly detection systems play a crucial role in enhancing energy efficiency by using smart sensors and sub-meters in residential buildings to detect abnormal power consumption patterns, thereby enabling proactive measures to reduce energy wastage and ensure sustainable energy practices [29,30,31,32]. Although extensive research is ongoing in IIoT, load forecasting, and anomaly detection, there is a significant gap concerning secure IIoT systems that incorporate both active and reactive load forecasting and anomaly detection. Specifically, this research highlights the need for simultaneous active and reactive load forecasting in a secure IIoT environment, emphasizing their significance in optimizing energy efficiency and enhancing predictive accuracy in industrial contexts. By addressing these challenges, the research aims to contribute to the development of more robust and comprehensive IIoT systems for sustainable energy management. The proposed system combines an optimized isolation forest model for real-time anomaly detection and a TCN-GRU-attention model for one-day-ahead load forecasting, achieving efficiency and reliability. The isolation forest model, ideal for resource-constrained SBCs like the Jetson Nano or mini PC, operates continuously by analyzing high-frequency data every second to detect anomalies promptly. Meanwhile, the TCN-GRU-attention model runs once daily via Windows Task Scheduler, reducing computational demands. This balanced approach ensures energy efficiency, computational optimization, and robust system performance in the IIoT framework.

### 1.1. Literature Review

The Industrial Internet of Things (IIoT) enhances monitoring and control systems by using AI for predictive analysis, thus minimizing downtime and optimizing efficiency. AI-driven analytics in energy management facilitates real-time adjustments, resulting in significant cost savings and reduced environmental impact. Advanced security protocols are essential to protect interconnected IIoT devices and data from cyber threats, ensuring the integrity and reliability of industrial operations. The integration of these technologies creates a cohesive ecosystem, enabling smarter, more resilient, and more sustainable industrial processes. In one study, the authors Mudaliar et al. [33] proposed an IoT-based real-time energy monitoring system using a Raspberry Pi, excluding aspects like AI-driven analytics, security, and protective measures. In a subsequent study, the authors Chen et al. [34] developed an IoT-enabled system for environmental and lighting control and monitoring using Node-RED, MQTT, and Modbus on an embedded Linux platform. This work presents a basic system focused on light status monitoring and control but does not address crucial aspects such as energy monitoring, data acquisition, AI-driven analytics, security, and protective systems. In a further study, the authors Gozuoglu et al. [35] proposed a CNN-LSTM-based deep learning application for load forecasting in a smart home environment using a Jetson Nano board. The research has significant limitations, including a lack of detail on security and protective measures, reactive load forecasting, and aspects related to edge and cloud servers. The research paper by Wang et al. [36] demonstrates a real-time electrical load forecasting and unsupervised anomaly detection framework, which shows promising results, and highlights its potential as an effective approach for electrical load forecasting in practice. However, the paper has some limitations, not addressing key aspects such as how data is fetched in real-time from the database, the real-time deployment of AI models, security measures, reactive load forecasting, or the integration of the real-time monitoring dashboards.

The authors Villegas-Ch et al. [37] introduce an AI-driven IoT system for real-time monitoring and predictive analytics in industrial environments. Their study’s notable limitations involve its remote monitoring capabilities and security measures, particularly concerning the transmission of data from the central server. Enhancing these aspects would be crucial to ensuring secure and efficient remote operations. The authors Sadeeq and Zeebaree [38] present a distributed IoT-based energy management system using ESP32 microcontrollers, demonstrating energy consumption reductions and data collection for an energy management system. The study’s shortcomings include challenges in accurately monitoring and measuring reactive power, along with significant security concerns related to potential vulnerabilities in data transmission and storage within the IoT-based energy management system. The advanced home energy management system presented by the authors Kumar et al. [39] is designed specifically for residential households in a microgrid environment. It integrates a smart energy meter to optimize energy usage based on real-time data, offering cost-effectiveness while accommodating user preferences and appliance-specific scheduling. While the research demonstrates promising results via simulation, practical implementation in real-life scenarios may face challenges related to integrating IoT and AI simultaneously, potentially impacting the system’s scalability and real-world applicability. The authors Rao et al. [40] underscore the role of smart energy management systems (SEMS) in optimizing energy consumption, mitigating peak demand challenges, and enhancing grid stability through algorithms and IoT integration. Their research has notable limitations concerning its lack of a real-time monitoring dashboard and insufficient detail on security measures and database management, particularly concerning edge and cloud servers. These aspects are critical for ensuring comprehensive oversight and protecting sensitive data in modern energy management systems.

In the realm of the Industrial Internet of Things (IIoT), active and reactive load forecasting plays a crucial role in anticipating and managing energy demands effectively. In the modern energy landscape, simultaneous active and reactive load forecasting is crucial to ensure grid stability and optimize resource management [41]. Active power forecasting enables the efficient distribution of real-time energy supply, while reactive power forecasting maintains voltage stability and enhances power quality. Together, these forecasts support the integration of renewable energy sources, manage dynamic consumer demands, and mitigate operational risks, all of which are essential for sustainable and resilient power systems in the face of evolving energy challenges [42]. Concurrently, anomaly detection mechanisms within the IIoT infrastructure monitor real-time data streams for irregularities or deviations from expected patterns. Real-time detection capabilities are crucial for promptly identifying potential equipment failures, operational inefficiencies, and abnormalities, ensuring timely notifications to users. The integration of load forecasting and anomaly detection capabilities within an IIoT ecosystem establishes a robust framework for fostering sustainable energy practices and ensuring reliable industrial operations. The authors Liu and Liang [43] introduce the AC-BiLSTM model, integrating convolutional neural networks (CNN), bidirectional long short-term memory networks (BiLSTM), and attention mechanisms for ultra-short-term power load forecasting, demonstrating enhanced stability in power systems. The drawbacks of the research outlined include its primary focus on active power forecasting without addressing reactive power forecasting and its lack of discussion on real-life integration of the AI model, such as implementation challenges, scalability issues, and practical deployment considerations. Furthermore, many existing load forecasting studies are constrained by their reliance on conventional AI models, which struggle to efficiently capture complex, non-linear relationships in load data and often overlook reactive load forecasting. Additionally, these studies often neglect to address real-life integration challenges, such as creating environments suitable for AI model implementation [26,44,45,46,47,48].

A machine learning-based approach for anomaly detection in electrical consumption profiles was presented by Luna-Romero et al. [49]. The study demonstrates a relative improvement in accuracy for anomaly detection but falls short in detailing the implementation of the ML model on a single-board computers (SBCs). This omission raises concerns about the model’s efficiency in such environments, including issues related to model size and other practical aspects of deployment. Several recent studies have introduced various anomaly detection methods, such as reconstruction-based frameworks with autoencoders [50], deep anomaly detection (DAD) [51], CNN-LSTM VAE [52], sparse U-net [53], conventional models [54], and LSTM-autoencoder [55]. However, these approaches often lack details on the computational efficiency of AI models, real-time deployment on SBCs, and other essential processes like real-time database fetching and visualization. Furthermore, many studies on anomaly detection in power consumption data overlook the transient conditions of appliances, such as refrigerators, and their impact on overall power usage [50,51,56]. Existing research has significant gaps in exploring real-time active and reactive load forecasting integrated with anomaly detection and protective systems within a secure IIoT environment. This study proposes an effective approach to implement these functionalities in real-time using both edge and cloud servers. Additionally, it introduces the utilization of single-board computers (SBCs) for implementing AI models.

### 1.2. Contribution

In their study, the authors Zhang et al. [42] proposed a dual-input LSTM model for forecasting reactive power loads to enhance power grid optimization, while in their own study, the authors Qin et al. [41] present a multi-task attention-LSTM (MTAL) model for forecasting both active and reactive power loads in power systems. In another study, the authors Mubarak et al. [57] introduce a stacked ensemble approach for forecasting active and reactive energy consumption in the steel industry. In a further study, the authors Dong et al. [58] propose a transformer-based deep learning model for short-term residential reactive power forecasting, using active power demand to enhance performance. These studies, while presenting different approaches for active and reactive load forecasting, have notable limitations. Some of the research relies on conventional AI models [41,42], which are susceptible to overfitting, potentially compromising their robustness and generalizability. Conversely, other studies employ more complex AI models [57,58] that increase computational demands and implementation complexity. Additionally, these studies often lack detailed discussions on the real-time deployment of forecasting AI models and the implementation of anomaly detection on SBCs, which is essential for practical applications in resource-constrained environments. Furthermore, there is a gap in addressing the data acquisition process, security, and the development of visualization dashboards, all of which are essential for effective energy management and decision-making in real-world scenarios. On the other hand, while some studies [56,59] propose IIoT platforms, they often overlook the integration of real-time active and reactive load forecasting along with protective measures. To address these challenges, this research introduces an AI-driven approach for short-term active and reactive load forecasting with integrated anomaly detection, designed for deployment in a secure IIoT environment with practical applications. The core contributions of this research are listed below:

- Implementation of a comprehensive and secure IIoT framework that integrates smart data acquisition systems (SDAS) with real-time monitoring, control, and protective measures.
- Deployment of a MariaDB database using structured query language (SQL) queries integrated with phpMyAdmin for efficient management of heterogeneous big data.
- Proposal of an efficient AI model that simultaneously predicts active and reactive power, along with optimized isolation forest models for anomaly detection that consider transient conditions in power consumption.
- Implementation of a real-time load-forecasting AI model (TCN-GRU-attention) on a centralized PC (cloud server) and anomaly detection AI models (optimized isolation forest) on edge devices, including a mini PC and a single-board computer like the Jetson Nano.
- The innovative architecture incorporates advanced AI-driven analytics and robust security measures, enhancing operational efficiency, ensuring data integrity, and significantly improving energy efficiency and management.

The remaining parts of the paper are outlined as follows: Section 2 describes the proposed methodology. Section 3 represents the details of the experimental setup. In Section 4, we present and analyze the experimental results. Finally, Section 5 discusses the conclusion and future work.

## 2. Methodology

### 2.1. System Overview

**Figure 1:**
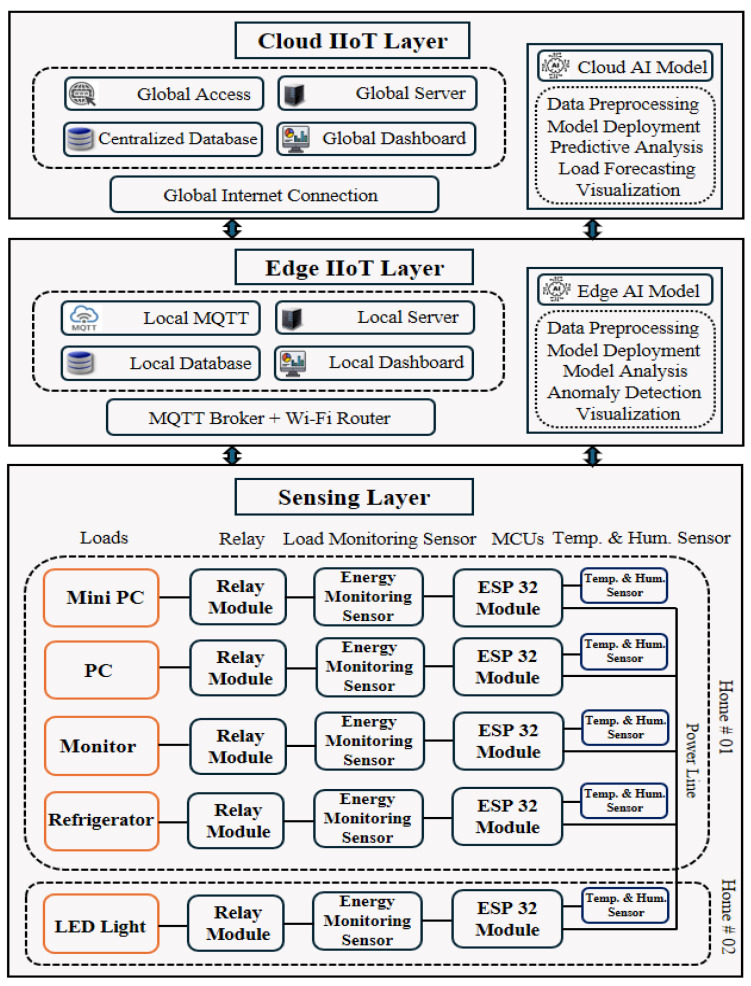
Proposed system architecture.

The proposed Industrial Internet of Things (IIoT) infrastructure consists of three essential parts: the sensing layer, the edge layer, and the cloud layer. The architecture of this system is illustrated in Figure 1, which depicts these three layers. In the sensing layer of the IIoT system, sensors and microcontrollers like the (Espressif Systems) ESP32 with temperature- and energy-monitoring (PZEM 004T) sensors gather real-time data from diverse industrial loads, including a mini PC, PC, refrigerator, and LED light. The data collected by the sensing layer are transmitted to the edge IIoT layer, where a local message queuing telemetry transport (MQTT) broker, a local server, and a local database handle real-time data processing and storage. This layer handles real-time data preprocessing, analyzes the edge AI model for anomaly detection, and visualizes any detected anomalies in the local dashboard. It ensures low-latency, highly reliable, lightweight, and secure data communication by utilizing MQTT brokers with the MQTT protocol over the wireless fidelity (Wi-Fi) routers. Finally, the edge IIoT Layer connects with the cloud IIoT layer or the centralized IIoT layer, which features a centralized database, global server, and dashboard for comprehensive data visualization and global access. This layer facilitates data preprocessing, AI model deployment, predictive analysis, and load forecasting, all supported by a global internet connection that ensures reliable and uninterrupted remote access to the cloud.

In this context, the Node-RED dashboard is employed to provide comprehensive real-time monitoring and data visualization, allowing users to continuously observe and interact with live data and trends, thereby enhancing the ability to make informed decisions based on current information. Overall, this is a process by which sensors collect environmental data and send them to an ESP32 module, which processes and publishes the data to an MQTT broker under a specific topic. The MQTT broker, acting as an intermediary, facilitates the distribution of these data to subscribers. Node-RED, subscribed to the relevant MQTT topic, receives and visualizes the data on its dashboard. Additionally, the data are stored in a database for historical data analysis, and AI models can analyze the stored data to make predictions and detect anomalies. This system offers real-time monitoring, stores historical data, performs advanced data analysis, and enhances decision-making capabilities.

### 2.2. Sensing Layer

The proposed architecture integrates IoT technologies with relay modules, load monitoring sensors, temperature and humidity sensors, and microcontrollers to efficiently monitor, control, and optimize energy usage and environmental conditions. Figure S1, detailed in the Supplementary Materials, illustrates a comprehensive energy management system implemented in two distinct homes, which are designated as Home 01 and Home 02. Home 01 includes various electrical loads such as a mini PC, PC, monitor, and refrigerator, while Home 02 currently includes a single electrical load, an LED light, with the potential to add other appliances as well. Each home is equipped with its own set of microcontrollers and sensors, ensuring that energy consumption and environmental data are independently monitored and managed. The structured layout of the system, with distinct pathways for each load type, ensures organized and efficient data management. Overall, sensors gather environmental data and transmit them to the ESP32 module, which then processes the information and publishes it to an MQTT broker under a designated topic.

Algorithm S1, detailed in the Supplementary Materials, establishes a secure WiFi and MQTT connection, reads sensor data, and publishes it to an MQTT topic. The setup initializes the WiFi with the given SSID and password, configures the MQTT server using the server address, username, password, and CA certificate, and sets a callback to handle messages on the subscription topic (*sub*). Table S1, also detailed in the Supplementary Materials, presents the common IoT devices used in this IIoT infrastructure, providing detailed descriptions of each device.

### 2.3. Edge IIoT Layer

**Figure 2:**
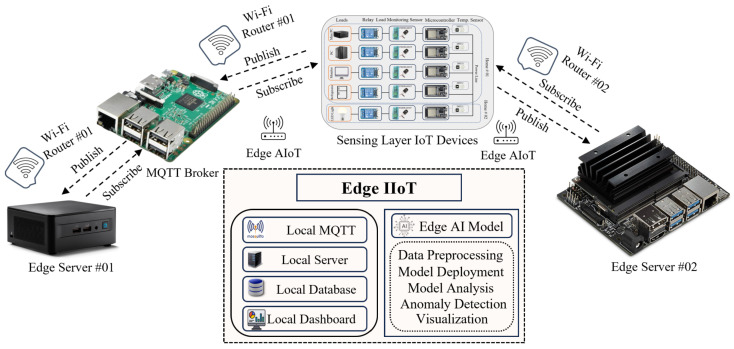
Proposed architecture for the edge IIoT layer.

Figure 2 illustrates a sophisticated layout for an edge-computing architecture designed to integrate IIoT with Artificial intelligence of things (AIoT) capabilities. It incorporates multiple interconnected components to facilitate real-time data processing, anomaly detection, and intelligent decision-making. At the core of this system are two edge servers, Edge Server 01 and Edge Server 02, which are connected through Wi-Fi routers (Router 01 and Router 02) and communicate using the MQTT protocol. These edge servers perform local data processing and host edge AI models for tasks such as data preprocessing, model deployment, model analysis, anomaly detection, and visualization.

Edge Server 01 is designed with a Raspberry Pi and a mini PC. The Raspberry Pi functions as a local MQTT broker, while the mini PC serves as the local machine for the edge server. The MQTT broker facilitates bi-directional communication (publishing and subscribing) with the IoT devices in the sensing layer and the mini PC, all through Wi-Fi Router 01. The mini PC in Edge Server 01 features AI capabilities for performing sophisticated data analysis and anomaly detection, enhancing the system’s ability to identify and respond to critical issues in real-time. Meanwhile, Edge Server 02, depicted as a Jetson Nano, is connected via Wi-Fi Router 02 and also acts as an MQTT broker. This Edge Server 02, likely equipped with an AI processing capability, focuses on data processing tasks such as model analysis, anomaly detection, and visualization. Both edge servers are connected to MariaDB databases for data storage, have AI capabilities for processing and analysis, and have Node-RED dashboards for comprehensive data management and real-time data visualization. Overall, this architecture enables local edge servers and efficient data handling, enhances data privacy, minimizes latency, and improves the responsiveness of the system. The process of deploying the AI model onto the mini PC and Jetson Nano begins with retrieving data from the MariaDB database. These data are then preprocessed to prepare them for analysis. Subsequently, an anomaly detection model is trained using the processed data. Once the model is effectively trained, it is deployed to the mini PC and Jetson Nano, where it is used to identify and detect anomalies. By deploying AI models at the edge, the system can quickly analyze and respond to anomalies or other critical events, ensuring reliable and efficient operation in industrial environments.

### 2.4. Centralized IIoT Layer

**Figure 3:**
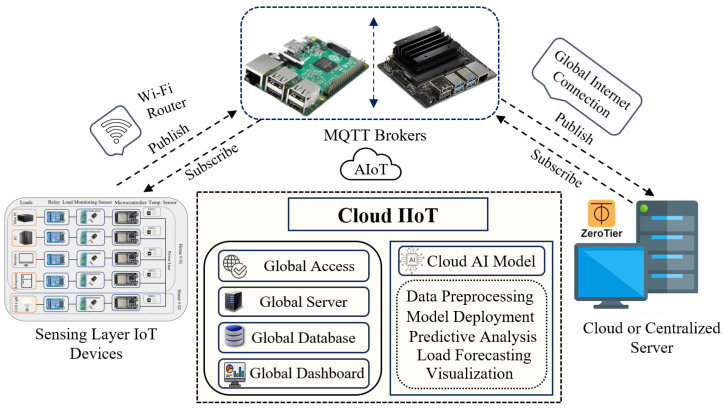
Proposed architecture for the cloud IIoT layer.

To centrally control and monitor the entire IIoT system, we have implemented a cloud or centralized IIoT architecture. Figure 3 illustrates a comprehensive architecture for an IIoT system integrated with artificial intelligence of things (AIoT) capabilities, using MQTT brokers for communication. At the foundational level, the sensing layer consists of various IoT devices, including load monitoring sensors, relays, microcontrollers, and temperature sensors, as we already discussed. The IoT devices gather data and transmit them to MQTT brokers through a Wi-Fi router. The MQTT brokers, depicted with images of single-board computers like the Raspberry Pi and Nvidia Jetson Nano, serve as intermediaries facilitating data exchange between the sensing layer and the cloud infrastructure. 

A global internet connection is used to transfer data from the MQTT brokers to centralized IIoT, enabling uninterrupted communication  with the cloud IIoT or centralized IIoT platform. The cloud IIoT platform comprises several key components—Global Access, Global Server, Global Database, and Global Dashboard—all of which are installed on a centralized server PC. These components ensure worldwide data access, secure data storage, and a user-friendly interface for monitoring and management. Furthermore, the cloud AI model, an integral part of the cloud IIoT, is responsible for advanced data processing tasks such as preprocessing, model deployment, predictive analysis, load forecasting, and visualization. The process involves fetching data from the MariaDB database, followed by preprocessing to prepare the data for analysis. Next, a load forecasting AI model is trained using these preprocessed data. Once the model is successfully trained, it is deployed for use, and finally, it generates forecasts to predict load demand. The data flow is bidirectional, with the cloud infrastructure capable of sending commands or data back to the MQTT brokers, which in turn, can communicate with the sensing layer devices. This closed-loop communication ensures real-time monitoring and control of the IoT devices, enabling efficient management of industrial processes. Additionally, ZeroTier is employed to securely [60] access the centralized IIoT or cloud IIoT server by creating a virtual network that ensures encrypted communication between remote devices and the server. This virtual network allows devices to connect directly to the cloud IIoT server over the global internet, bypassing the potential security risks associated with traditional network setups. By using ZeroTier’s secure peer-to-peer architecture, we can facilitate smooth and reliable access to the IIoT platform for efficient real-time monitoring and data management. 

### 2.5. Implementation of AI Models

Current research in the Industrial Internet of Things (IIoT) focuses on enhancing energy management systems with real-time monitoring and data analytics to improve system efficiency and reduce costs. AI models for load forecasting and anomaly detection are becoming more advanced, using deep learning to accurately predict demand patterns and identify system irregularities. However, challenges persist, including the integration complexity, data privacy concerns, and the need for substantial computational resources to train and deploy these models effectively. Our IIoT infrastructure has successfully addressed these challenges by effectively training and deploying AI models in both the edge and the cloud IIoT environment, ensuring robust performance across the entire architecture.

#### 2.5.1. Active and Reactive Load Forecasting

To train the AI model (TCN-GRU-attention), it is essential to establish a connection with the database to gather data directly from it in real-time. Without establishing a direct connection to the database, the procedure falls back on the conventional approach of loading data from local machines, which is inherently less efficient and effective. This traditional method, lacking real-time capabilities, significantly hampers the effectiveness of the training process and fails to fully utilize the potential of dynamic data processing and optimization. Algorithm S2, detailed in the Supplementary Materials, effectively demonstrates how to connect to a MariaDB database, retrieve a large dataset, and load it into a Pandas DataFrame for each specified table.

**Figure 4:**
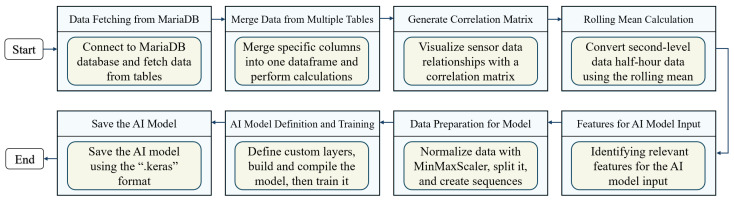
A comprehensive workflow for developing a load forecasting AI model.

Figure 4 presents a detailed workflow for developing an AI model using sensor data, organized into several critical stages. It begins with data fetching from MariaDB, where data are extracted from a database to form the foundation for analysis. Next, in the process of merging data from multiple tables, specific columns are combined into a single dataframe. This ensures comprehensive calculations, such as computing the total power consumption. The workflow progresses to generate a correlation matrix, visualizing sensor data relationships in order to identify significant correlations for feature selection and model accuracy.

In the rolling mean calculation stage, second-level data are converted to half-hour data, which is essential for short-term load forecasting. Following this, for the AI model input, relevant features such as temperature, humidity, voltage, active power, and reactive power have been selected to capture key factors influencing performance and accuracy. During data preparation for the model, the data are normalized, split into training and testing datasets, and sequenced to ensure they are in the right format for model training. Table 1 presents the hyperparameters applied in the proposed model. During AI model definition and training, custom layers are defined, and the model is built, compiled, and trained with the prepared data. The process concludes with saving the AI model, during which the trained model is saved in the *.keras* format for future use and integration.

#### 2.5.2. Anomaly Detection

Anomaly detection in smart energy management systems is crucial for identifying and mitigating irregularities that could lead to energy inefficiencies and system failures, ensuring optimal performance and reliability. In our proposed system, AI models are employed for anomaly detection to provide critical insights into potential threats, ensuring the identification and resolution of irregularities in smart energy management systems.

**Figure 5:**
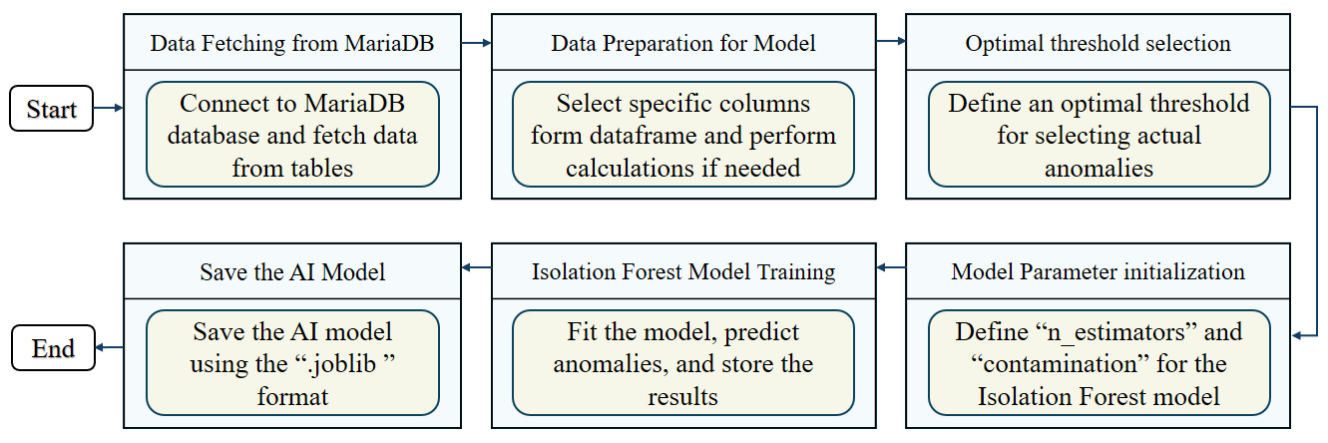
A comprehensive workflow for developing an anomaly detection AI model.

Figure 5 depicts a structured workflow for developing an AI model aimed at detecting anomalies, particularly using the isolation forest model. The process begins with data fetching from MariaDB, which involves connecting to a MariaDB database to retrieve essential data from various tables. This initial step is fundamental, as it provides the raw data required for the entire analysis. Following data acquisition, the data preparation for the model stage involves selecting specific columns from the dataframe and performing any necessary calculations. This step ensures that the data are in the correct format and that they contain all the relevant information needed for anomaly detection. Next, in the optimal threshold selection phase, an optimal threshold is defined for selecting actual anomalies. This is a critical step, as it determines the sensitivity of the model in detecting anomalies, balancing the trade-off between false positives and false negatives. To optimize the isolation forest model for anomaly detection under transient conditions of electrical appliances, we performed a hyperparameter tuning process by using grid search.

The workflow then moves to the model parameter initialization stage, where key parameters such as *n_estimators* and *contamination* for the isolation forest model are defined. Table 2 presents the hyperparameters of the proposed isolation forest model. The thresholds are selected to exceed the normal power consumption by the appliances, based on data provided by the appliance model and manufacturer. The total power consumption threshold is set higher than the overall power consumption during normal operating conditions. These thresholds have been carefully selected to ensure accurate detection and performance of the model. These parameters are crucial for configuring the model to accurately identify anomalies in the dataset. In the isolation forest model training phase, the model is fitted with the prepared data, and anomalies are predicted and stored. This stage represents the core of the anomaly detection process, where the model learns patterns from the data and identifies outliers. Finally, the save the AI model step involves saving the trained AI model using the *.joblib* format. This ensures that the model can be easily reused and deployed in different environments for continuous anomaly detection.

### 2.6. Model Description

This section provides a detailed description of the AI models used in this study, including the load forecasting AI model and the anomaly detection AI model, along with an overview of their overall structure and functionality.

#### 2.6.1. Load Forecasting Model

In this proposed method, we introduce a hybrid deep learning model that goes beyond traditional methods which typically rely on conventional neural networks or statistical approaches. Our model integrates advanced techniques, including temporal convolutional network (TCN), gated recurrent units (GRU), and an attention mechanism to achieve enhanced performance. The TCN-GRU-attention model effectively addresses the unique characteristics of active and reactive loads through a combination of TCNs, GRUs, and an attention mechanism. TCNs capture long-term dependencies, GRUs efficiently manage sequential patterns, and the attention mechanism focuses on key patterns in each load type. This integrated approach enables the model to accurately predict both active and reactive loads, effectively capturing the cyclic behavior of active and reactive loads. The model is composed of three TCN blocks, three GRU layers, and an attention layer.

**Figure 6:**
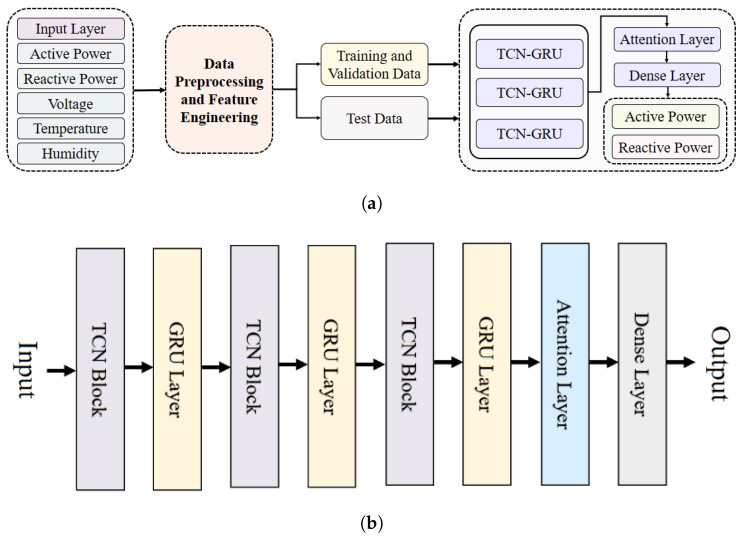
(**a**) illustrates the overall model architecture, while (**b**) shows the arrangement of the TCN and GRU layers.

Figure 6a depicts the overall model architecture, while Figure 6b presents the arrangement of the TCN and GRU layers along with an attention mechanism and a dense layer. This model presents a novel approach by simultaneously forecasting both active and reactive power loads, which is crucial for modern energy distribution and management systems. This capability addresses a significant gap in existing technologies, which often only concentrate on active power forecasting and overlook the critical role of reactive power in grid stability and energy efficiency.

The temporal convolutional network (TCN) is highly effective for load forecasting because it captures long-range dependencies while maintaining causality in sequence modeling [61,62]. Its architecture is designed to efficiently process varying lengths of historical data, which is crucial for predicting electrical load patterns that rely on past behaviors over extended periods. This ability ensures that forecasts are grounded in relevant historical contexts, resulting in more accurate and reliable predictions essential for energy management and planning [63,64].

**Figure 7:**
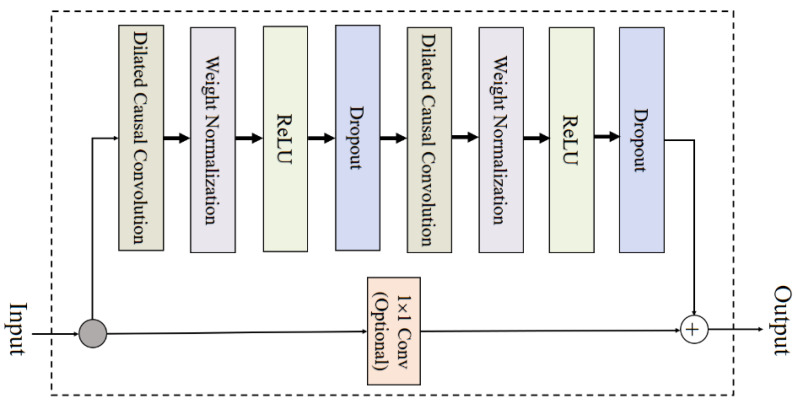
A single TCN block architecture.

As shown in Figure 7, the temporal convolutional network (TCN) block uses the dilated causal convolution method illustrated in Figure S2 to effectively handle sequence modeling tasks, as detailed in the Supplementary Materials. This approach is particularly powerful for tasks where learning from long-range dependencies is crucial. The architecture’s primary feature is the dilated causal convolution, ensuring that the output at any time *t* is computed only from current and previous time steps, preserving temporal causality. Other key features include dropout layers to prevent overfitting, weight normalization for stable training, and ReLU activation, which allows the network to learn complex patterns in the data. An optional 1 × 1 *convolution* can be used to align dimensions for residual connections. At the core of the TCN is the dilated causal convolution, which is mathematically represented in Equation (1).

(1) $$y_{t}=\sum_{k=0}^{K-1}f_{k}\cdot x_{t-d\times k}$$

where $y_{t}$ is the output before applying a non-linear activation function such as *ReLU*, $f_{k}$ are the filter weights, $x_{t-d\times k}$ are the inputs influenced by the dilation factor *d*, and *K* is the filter size. Additionally, the residual connection is represented in Equation (2).

(2) $$Y_{\mathrm{out}}=\mathrm{ReLU}\left(y_{t}\right)+\mathrm{Conv}1D\left(x_{t}\right)$$

where $x_{t}$ represents the input to the block, which is added to the output of the convolution to form a residual connection, and $Y_{\mathrm{out}}$ is the final output after the ReLU activation and potential dropout regularization. The output of the dilated causal convolution is often added back to the original input, using a convolutional layer to adjust dimensions. This process facilitates deeper networks by preventing the vanishing gradient problem and allowing both the original input and the learned features to propagate through the network.

A gated recurrent unit (GRU) model is crucial for load forecasting due to its ability to efficiently capture and process sequential dependencies in time-series data, enabling accurate predictions. It addresses the vanishing gradient problem more efficiently than traditional RNNs, enabling it to learn from data points that occurred long ago in the sequence, which is vital for predicting future trends based on past patterns [65]. GRU is computationally efficient compared to long short-term memory (LSTM) models because it uses fewer parameters [66,67]. This efficiency not only accelerates the training process but also reduces computational overhead, making GRU suitable for environments with limited computational resources.

**Figure 8:**
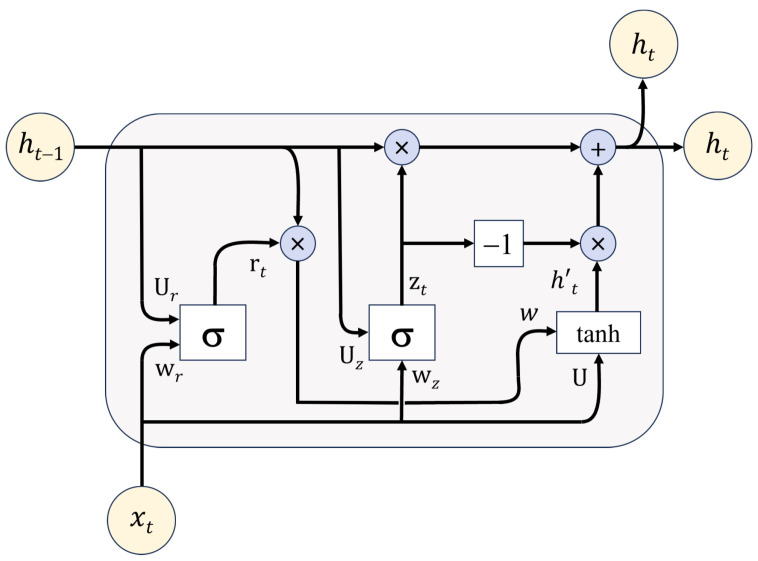
The architecture of a single GRU layer.

Moreover, the architecture of GRU, featuring update and reset gates, allows for flexible and robust modeling of time series data. These gates effectively control the flow of information, enabling GRU to dynamically retain or forget information. This adaptability is especially beneficial for load forecasting, where sudden changes in usage patterns can significantly influence prediction accuracy. The architecture of the GRU model is illustrated in Figure 8.

Here are the equations that describe each step in the GRU mechanism:

(3) $$z_{t}=\sigma \left(W_{z}\cdot \left[h_{t-1},x_{t}\right]\right)$$

(4) $$r_{t}=\sigma \left(W_{r}\cdot \left[h_{t-1},x_{t}\right]\right)$$

(5) $${\tilde{h}}_{t}=\tanh\left(W\cdot \left[r_{t}⊙h_{t-1},x_{t}\right]\right)$$

(6) $$h_{t}=\left(1-z_{t}\right)⊙h_{t-1}+z_{t}⊙{\tilde{h}}_{t}$$

The GRU operations involve several key components. The gates $r_{t}$ and $z_{t}$ process inputs $x_{t}$ (current input) and $h_{t-1}$ (previous hidden state) through sigmoid functions (σ), determining the flow of information and state updates. The candidate hidden state ${\tilde{h}}_{t}$ is generated by applying the hyperbolic tangent function tanh to a mixture of the current input and the gated previous hidden state, introducing non-linearity. Finally, the new hidden state $h_{t}$ is derived by interpolating between the previous state $h_{t-1}$ and the candidate state ${\tilde{h}}_{t}$, with the interpolation weighted by the update gate $z_{t}$, effectively updating the state with a balance of past and new information.

The attention mechanism is a method that mimics the human brain’s ability to selectively focus on important aspects of observed information based on the task’s needs while ignoring irrelevant parts [68]. Figure S3, detailed in the Supplementary Materials, presents the architecture of an attention layer along with an overview of its functionality. In sequence-to-sequence modeling tasks, this allows models to dynamically concentrate on key elements of the input sequence, enhancing the accuracy and context-awareness of the output. This process involves calculating attention scores to determine the importance of each input feature, converting these scores into attention weights using a softmax function, and forming a context vector by taking a weighted sum of the input features [69]. The attention layer’s weights are calculated through the following process:

(7) $$u_{t}=\mathrm{utanh}\left(wh_{t}+b\right)$$

(8) $$\alpha_{t}=\frac{\exp(e_{t})}{\sum_{j=1}^{t}e_{j}}$$

(9) $$y_{t}=\sum_{t=1}^{i}\alpha_{t}h_{t}$$

The attention-scoring function calculates the correlation between the input features and the output vector of the GRU layer at a given time. The weight coefficients are represented by *u* and *w*, while the bias coefficient is denoted by *b*. $\alpha_{t}$ is the attention probability [69,70].

#### 2.6.2. Anomaly Detection Model

Isolation forest is an anomaly detection algorithm that identifies outliers by isolating data points [71]. It constructs an ensemble of random trees, where each tree isolates data points by recursively selecting a feature and a random split value. Anomalies are isolated more quickly, resulting in shorter average path lengths in the trees. The anomaly score for each point is based on this average path length, with shorter paths indicating a higher likelihood of being an anomaly. Compared to traditional algorithms like local outlier detection and K-means, the isolation forest algorithm is more robust for handling high-dimensional data [72]. It identifies anomalies by isolating data points in fewer splits, with anomalies (simple or complex) being easier to isolate due to their rarity. Unlike simple outliers, complex anomalies are isolated more effectively because they occupy sparse regions in the data, making them distinct from normal behavior. Here are the key mathematical operations involved in the isolation forest anomaly detection model:

(10) $$s\left(x,n\right)=2^{-\frac{E(h(x))}{c(n)}}$$

where E(h(x)) is the expected path length of *x*, and c(n) is the average path length of unsuccessful searches in a binary search tree, approximated as:

(11) $$c\left(n\right)=2H\left(n-1\right)-\left(\frac{2(n-1)}{n}\right)$$

with H(i) as the harmonic number, defined as:

(12) H(i)=ln(i)+γ

where γ is the Euler constant [72].

#### 2.6.3. Evaluation Metrics

Evaluation metrics are essential for load forecasting models as they provide quantitative measures to assess the accuracy and reliability of the model’s predictions. They help by comparing different models and selecting the best-performing one for practical applications. Common evaluation metrics include the mean absolute error (MAE), mean squared error (MSE), and root mean squared error (RMSE). These metrics offer insights into the average magnitude of errors, the squared average of these errors to emphasize larger deviations, and the square root of the average squared errors for interpretability in the original units, respectively. The use of these metrics ensures that models are robust, accurate, and applicable to real-world scenarios. The mathematical equations for these evaluation metrics are as follows:

(13) $$\mathrm{MAE}=\frac{1}{n}\sum_{i=1}^{n}\left|y_{i}-{\hat{y}}_{i}\right|$$

(14) $$\mathrm{MSE}=\frac{1}{n}\sum_{i=1}^{n}{\left(y_{i}-{\hat{y}}_{i}\right)}^{2}$$

(15) $$\mathrm{RMSE}=\sqrt{\frac{1}{n}\sum_{i=1}^{n}{\left(y_{i}-{\hat{y}}_{i}\right)}^{2}}$$

where $y_{i}$ is the actual load, ${\hat{y}}_{i}$ is the predicted load, and *n* is the number of observations.

Evaluation metrics are also important for evaluating the performance of anomaly detection models like the isolation forest. They help to determine how effectively the model identifies anomalies. Key metrics include precision, recall, F1 score, and accuracy. Precision measures the proportion of correctly identified anomalies among all instances classified as anomalies. Recall measures the proportion of correctly identified anomalies among all actual anomalies. The F1 score, as the harmonic mean of precision and recall, balances these two metrics. Accuracy measures the proportion of correctly classified instances overall. These metrics help ensure the model’s effectiveness and reliability in practical applications. The mathematical equations for these evaluation metrics are as follows:

(16) $$\mathrm{Precision}=\frac{TP}{TP+FP}$$

(17) $$\mathrm{Recall}=\frac{TP}{TP+FN}$$

(18) $$F1\;\mathrm{Score}=2\cdot \frac{\mathrm{Precision}\times \mathrm{Recall}}{\mathrm{Precision}+\mathrm{Recall}}$$

(19) $$\mathrm{Accuracy}=\frac{TP+TN}{TP+TN+FP+FN}$$

where TP is the number of true positives, FP is the number of false positives, TN is the number of true negatives, and FN is the number of false negatives.

### 2.7. Securing the Overall IIoT Infrastructure

In the realm of the Industrial Internet of Things (IIoT), security concerns are frequently overlooked, leading to significant vulnerabilities. The numerous connected devices, often lacking robust security measures, are especially susceptible to serious threats such as unauthorized access, data breaches, and cyberattacks. These threats compromise the integrity and functionality of essential industrial systems, highlighting the critical need for enhanced security protocols to protect against potential disruptions and data compromises. In this proposed system, we ensure enhanced security measures by providing robust protection for the MQTT broker, Node-RED server, and MariaDB database, along with secure remote access. Our approach includes encryption, data integrity, and user authentication, significantly enhancing the reliability and acceptance of the overall IIoT environment.

**Figure 9:**
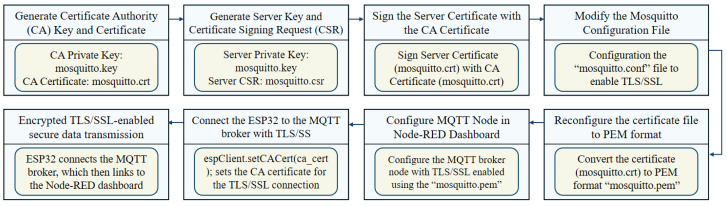
A detailed process for securing an MQTT broker.

The security process for the MQTT broker begins with configuring Mosquitto to use username and password authentication. Using the Mosquitto password file utility, a password file is created for users, ensuring that only authenticated clients can connect. The Mosquitto configuration file is then modified to reference this password file and to disable anonymous connections, enhancing the security of the broker. Figure 9 outlines a comprehensive process for securing an MQTT broker using TLS/SSL, connecting it to the Node-RED dashboard, and integrating it with an ESP32 device. The process starts with generating a certificate authority (CA) key and certificate, followed by creating a server key and certificate signing request (CSR). The server certificate is then signed with the CA certificate, ensuring trusted communication. The Mosquitto configuration file is modified to enable TLS/SSL, referencing the necessary certificates and keys. The certificate is converted to *PEM* format for compatibility, and the MQTT node in Node-RED is configured to use this secured connection. The final steps involve configuring the ESP32 to connect to the MQTT broker using the CA certificate for TLS/SSL encryption, ensuring secure data transmission. By setting the CA certificate in the ESP32 code *(espClient.setCACert(ca_cert))*, a secure, encrypted connection is established. This setup guarantees that all data transmitted between the ESP32, MQTT broker, and Node-RED dashboard are encrypted, maintaining data integrity and confidentiality. This process ensures robust security for IIoT applications by protecting against unauthorized access and data breaches.

**Figure 10:**
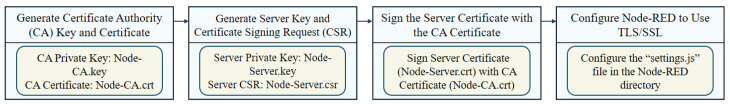
A comprehensive process for securing the Node-RED server.

To secure the Node-RED server, it is crucial to generate TLS/SSL certificates using *OpenSSL* to enable Hypertext Transfer Protocol Secure (HTTPS), ensuring encrypted communication. The process illustrated in Figure 10, which involves setting up a self-signed certificate authority (CA) and server certificates on a local machine, is somewhat complex, but it is essential for securing the Node-RED server. Initially, the CA’s private key and certificate are created using *OpenSSL* commands. Subsequently, a private key and a certificate signing request (CSR) for the server are generated. This CSR is then signed with the CA certificate to produce the server certificate. Finally, adjustments are made in the *settings.js* file of Node-RED to enable HTTPS, incorporating the newly created certificates. By following this process, enhanced security through encrypted communication is achieved for the Node-RED server. Additionally, a *bcrypt* hash for the desired password is generated, and the *settings.js* file is updated to include *adminAuth* settings, specifying the username and hashed password. The changes are saved, and Node-RED is restarted to apply the new configurations. Finally, the setup is verified by accessing the Node-RED dashboard via HTTPS protocol and logging in with the configured credentials. 

Securing the MariaDB and phpMyAdmin installations is essential to prevent unauthorized access and potential data breaches. Using the *mysql_ secure_installation* script ensures that the MariaDB server is secured by setting a strong root password, removing anonymous users, disabling remote root login, and eliminating unnecessary databases. This process minimizes vulnerabilities and restricts access to authenticated users only. For phpMyAdmin, ensuring security involves configuring Apache properly and creating a dedicated user with strong credentials instead of using the root account, which helps minimize the risk of unauthorized database access and potential misuse of administrative privileges. Lastly, for secure remote access to the cloud or centralized IIoT server, ZeroTier provides end-to-end encryption, ensuring that data remain protected from potential threats. Additionally, it uses software-defined networking principles to create isolated virtual networks, enhancing overall network security. Furthermore, Algorithm S1, described in the Supplementary Materials, establishes a secure WiFi connection and MQTT broker communication using encrypted credentials, the server address, and a CA certificate, ensuring encrypted communication for sensor data publishing and secure message handling.

## 3. Experimental Setup

This section presents a detailed overview of the experimental setup, highlighting the organization of the sensing, edge, and cloud layers in a structured manner.

### 3.1. Experimental Setup of Sensing Layer

The smart data acquisition system in Figure S4 (detailed in the Supplementary Materials) features a rigid and compact setup, which is both portable and secure. By enclosing all components within a sealed plastic case, it ensures maximum safety, preventing exposure to electrical parts and mitigating any risk of accidental contact or damage. The application of the smart data acquisition system (SDAS) is connected to various appliances—a mini PC, a PC, monitors, a refrigerator, and an LED light—each located in different rooms. In this context, we are referencing specific devices set up in different rooms for two homes: for Home 01 (Edge Server 01), we have designated the mini PC as Room 1, the PC as Room 2, the monitor as Room 3, and the refrigerator as Room 4. Meanwhile, for Home 02 (Edge Server 02), we have set up the LED light in Room 5. These appliances are controlled by relays, enabling remote control and protective functionality, which enhances safety and efficiency within the IIoT framework.

### 3.2. Experimental Setup of Edge IIoT Layer

**Figure 11:**
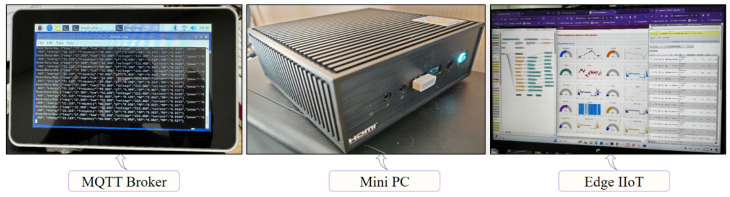
Configuration of the edge IIoT layer.

Figure 11 illustrates the core components of Edge Server 01 within an advanced edge-computing architecture designed for IIoT and AIoT integration. The left image displays a Raspberry Pi with a touchscreen display functioning as an MQTT broker, facilitating bi-directional communication between IoT devices and the mini PC through Wi-Fi Router 01. The central image features the mini PC, serving as the local machine of Edge Server 01, equipped with AI capabilities. This mini PC retrieves data in real-time from the MariaDB database installed on its local machine, preprocesses the data, and runs AI models for anomaly detection, thereby enabling real-time identification of irregularities and response to critical issues.

The right image shows the Node-RED dashboard installed in the mini PC, part of the edge IIoT interface, providing comprehensive data management and real-time visualization. The image on the right displays the Node-RED dashboard installed on the mini PC, which is a component of the Edge IIoT interface. This dashboard offers comprehensive data management, relay control options, and real-time visualization. This architecture ensures efficient data handling and reliable operation, using AI for data analysis and anomaly detection. 

### 3.3. Experimental Setup of Centralized IIoT Layer

**Figure 12:**
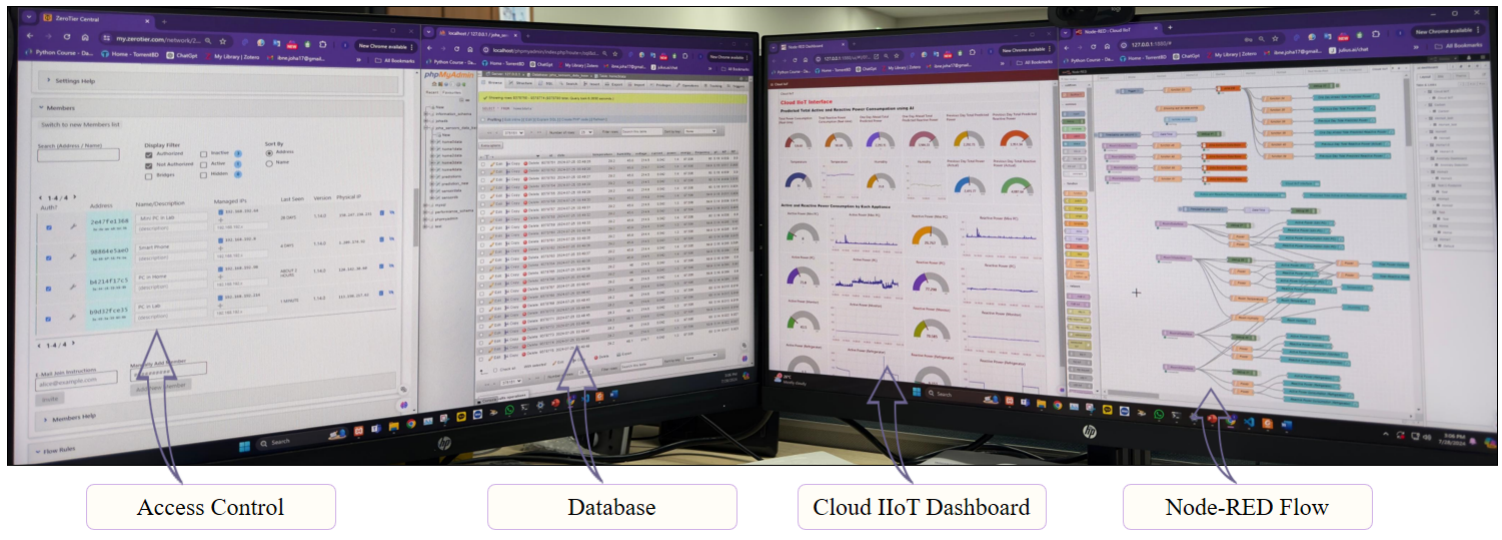
Configuration of the cloud or centralized IIoT layer.

The cloud IIoT interface provides real-time monitoring, advanced data analysis, and global accessibility, and for this reason, we need it to enable efficient and informed decision-making for industrial processes. The figure illustrates key components of an advanced Industrial Internet of Things (IIoT) system with integrated artificial intelligence of things (AIoT) capabilities, as shown in Figure 12. The left screen shows the ZeroTier interface, which manages network access and control, ensuring secure, encrypted communication between remote devices and the IIoT server. This interface allows for the management and monitoring of connected devices, enhancing network security. The second screen from the left shows the phpMyAdmin interface for the MariaDB database, displaying real-time data from IoT devices such as ESP32 to MQTT brokers. These brokers connect to Node-RED, which interfaces with the database. This setup allows for efficient data management, querying, and visualization, essential for AI model processing tasks like load forecasting.

The second screen from the right features the cloud IIoT Dashboard, providing visual representations of key performance indicators, real-time control options, and data trends. This dashboard enables real-time visualization of AI-processed data, facilitating decision-making with insights into load forecasting and thus enhancing system monitoring and management. The right screen shows the Node-RED interface, used for creating and managing data flows within the IIoT system. This interface displays the logical flow of data from IoT devices through MQTT brokers to the cloud infrastructure, integrating nodes for data collection, preprocessing, AI model execution, and output visualization. Node-RED supports the design and deployment of complex workflows, ensuring efficient data handling and real-time processing. To sum up, the figure illustrates the secure, efficient, and real-time capabilities of the IIoT system: ZeroTier ensures secure access, MariaDB manages data efficiently, the cloud IIoT dashboard facilitates real-time monitoring and control features, and Node-RED enables flexible workflow design, collectively optimizing industrial operations.

## 4. Experimental Results

This section provides a detailed discussion of the conducted experiments and their corresponding results.

### 4.1. Dataset Description

**Figure 13:**
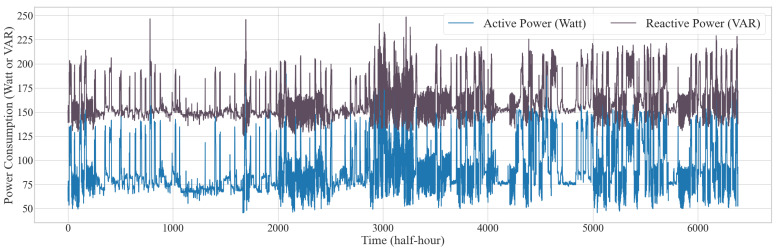
Visual representation of a historical active and reactive power consumption dataset.

In this study, real-life data is collected using weather and energy monitoring sensors, with the data continuously stored in a database for real-time analysis. To effectively train the AI model, it is crucial to connect directly to the database and retrieve data in real-time. Algorithm S1 already discussed the process of retrieving a large dataset and loading it into a Pandas DataFrame for each specified table. In the process outlined, we gathered data from multiple sources, including *room1data*, *room2data*, *room3data*, *room4data*, and *room5data*, and then preprocessed these data to ensure they were properly formatted and cleaned for subsequent analysis and model training. To perform load forecasting, we focused on the data from the first four rooms (Edge Server 01), where we retrieved 133 days’ worth of data recorded at one-second intervals. Using the data from the first four rooms, we calculated the total active and reactive power consumption, which primarily represents the overall energy usage for Home 01. Given the high frequency of the original dataset, we resampled the data to create half-hour (30-min) intervals to enhance the effectiveness and accuracy of the load forecasting process. This resampling process allows us to capture broader trends and patterns in the data while reducing noise, making it more suitable for forecasting future energy consumption with greater reliability. Figure 13 illustrates the total active and reactive power consumption for Home 01. This visualization provides a comprehensive overview of the energy usage patterns by aggregating the power consumption data from the mini PC, PC, monitor, and refrigerator. In the preprocessing stage, we selected temperature, humidity, voltage, active power, and reactive power as the input features, with active power and reactive power serving as the output features for the load forecasting AI model. Due to the strong positive correlation between active and reactive power, using active power to forecast reactive power, or vice versa, is not viable due to potential data leakage issues [58]. Therefore, we propose an AI model that simultaneously forecasts both active and reactive power demand.  During this process, we used 107 days of data for training the model, ensuring that it has a substantial historical basis to learn from. The remaining data were set aside for testing, allowing us to evaluate the model’s performance and accuracy in forecasting future energy loads.

For anomaly detection, we retrieved three months of data from the MariaDB database. We used 72 days of these data to train the isolation forest model for Edge Server 01 (Home 01), and the remaining 18 days were used to test the model. Furthermore, for Edge Server 02 (Home 02), we retrieved 12 days of data, where 9 days of data were for the model training, and used the subsequent 3 days of data for testing. Since we used a single-board computer (Jetson Nano) for Edge Server 02, we reduced the dataset size for training and testing to optimize processing while maintaining accuracy. By focusing on active power, the model can effectively identify unusual patterns and deviations from normal consumption behavior, which may indicate potential anomalies or irregularities in the operation of home appliances. Finally, we saved and evaluated the models to ensure their accuracy and reliability before deploying them for load forecasting and anomaly detection. One major issue during anomaly detection is data imbalance, which occurs when anomalies are much rarer than normal behavior, leading to models favoring the majority class (normal behavior) and reducing their ability to detect anomalies. To address this, we employed data labeling techniques, including random data generation, to create synthetic anomalous data points and achieve a balanced dataset. This approach enables the isolation forest model to more effectively learn the distinguishing characteristics of anomalies. The dataset used in this study, which includes electrical parameters and weather data for load forecasting and anomaly detection, is not publicly accessible due to privacy concerns. However, it can be made available upon reasonable request to the authors.

### 4.2. Performance Evaluation of AI Models

This section presents the performance evaluation of the AI models for load forecasting and anomaly detection.

#### 4.2.1. Active and Reactive Load Forecasting

The load forecasting AI model was trained using the dataset we previously described and was subsequently tested using several evaluation metrics. These metrics were selected to measure the reliability and performance of the AI model in real-world scenarios. Our evaluation focused on determining how well the model could predict active and reactive power demand in practical applications. The evaluation process involved comparing our model, TCN-GRU-attention, to other existing models in order to determine its superiority in forecasting accuracy and efficiency. In a research paper, the authors [41] introduce an approach for active and reactive load forecasting that uses the strengths of LSTM networks and attention mechanisms within a multi-task learning framework. This approach was compared to both the LSTM-attention model and other conventional models.

**Figure 14:**
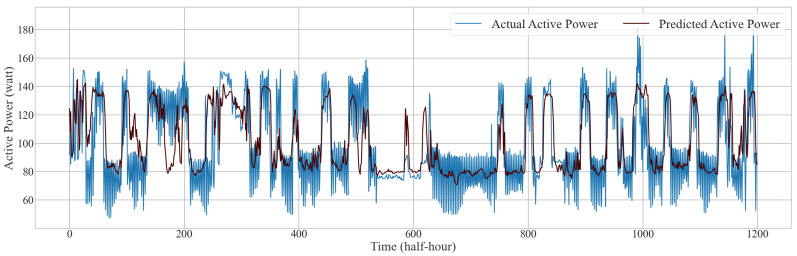
Actual versus predicted active power using the TCN-GRU-attention model.

**Figure 15:**
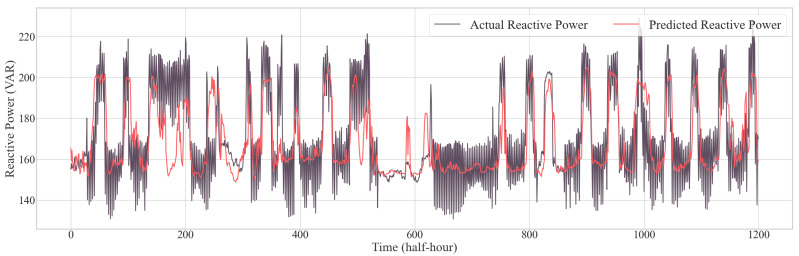
Actual versus predicted reactive power using the TCN-GRU-attention model.

In another study, the authors [35] use a CNN-LSTM model for active power forecasting. Similarly, in our study, we compared our model with these existing and other conventional models to evaluate its effectiveness. As shown in Table 3 and Table 4, the TCN-GRU-attention model consistently outperformed other models across several key metrics, demonstrating its ability to provide more accurate and reliable predictions of both active and reactive power demand. Figure 14 provides a visual representation of the actual versus predicted active power using the TCN-GRU-attention model, while Figure 15 presents a visual representation of the actual versus predicted reactive power using the same model. From these prediction curves, it is evident that this model is capable of simultaneously predicting both active and reactive power. The performance metrics for active load forecasting are as follows: MSE is 0.0183, MAE is 0.1022, and RMSE is 0.1354. For reactive load forecasting, the corresponding metrics are 0.0202 for MSE, 0.1077 for MAE, and 0.1422 for RMSE. This performance highlights the model’s potential for real-world applications, where precise load forecasting is crucial for optimizing energy management, grid stability, and energy efficiency.

#### 4.2.2. Anomaly Detection

The anomaly detection AI model was trained using the dataset we previously described and was tested with several evaluation metrics. These metrics were chosen to assess the model’s reliability and performance in real-world scenarios. Our evaluation aimed to determine how effectively the model could identify anomalies or unusual behavior in practical applications.

We propose optimized isolation forest models for anomaly detection that consider the transient conditions of appliances, such as refrigerators, and the impact of these transient effects on overall power consumption. Transients occur in appliances when they start up or shut down, causing brief electrical spikes due to sudden changes in electrical current flow. This condition is normal, as appliances like refrigerators and water dispensers are designed to manage brief electrical spikes during startup or shutdown.

Many studies overlook the transient fluctuations in electrical appliances, such as refrigerators and water dispensers, often misclassifying these normal variations as anomalies, while also not considering the complexities of real-time deployment [50,51,73]. Our method for detecting transients involves defining critical thresholds for power and rate-of-change over one, two, and three periods or time intervals in seconds. By checking if the power exceeds a certain level and whether any rate-of-change values surpass their respective thresholds, the method effectively distinguishes significant transients from normal fluctuations. This approach accurately identifies transient conditions, reducing false positives and improving the reliability of anomaly detection in power consumption data. We apply this method to detect anomalies in the power consumption data of refrigerators and total power consumption, as these exhibit transient conditions. For appliances in which no transients are detected, we employ the conventional isolation forest model with fine-tuned hyperparameters to identify anomalies.

The proposed isolation forest models have collectively demonstrated high effectiveness in detecting anomalies or unusual behavior within the IIoT system. Table 5 presents an analysis of the performance of the proposed anomaly detection models. Averaging the performance data across six models, the precision score is 0.95, indicating that 95% of the detected anomalies were indeed actual anomalies. This consistently high precision across models underscores their ability to minimize false positives, ensuring that normal behavior is not misclassified as an anomaly. The average recall score is 0.98, reflecting the models’ collective capability to identify 98% of the actual anomalies present in the data. This high recall across multiple models highlights their effectiveness in capturing most of the anomalies, thereby reducing the risk of missing critical issues within the system. The average F1 score, calculated as the harmonic mean of precision and recall, is 0.96. This balance between precision and recall across all models underscores their overall robustness and reliability in identifying anomalies without sacrificing accuracy. Moreover, the average accuracy of the models is close to 1, further emphasizing their effectiveness in correctly classifying both normal and anomalous behavior within the system. This high level of accuracy suggests that the ensemble of models is well-suited for real-world applications, providing reliable anomaly detection in complex IIoT environments. Our proposed isolation forest model demonstrated superior performance in anomaly detection for power consumption data, with better average performance metrics, while also addressing the transient conditions that the existing model overlooks [56].

**Figure 16:**
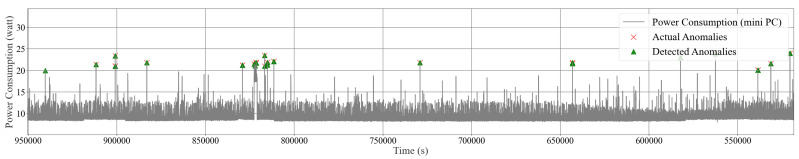
Detected anomalies in the mini PC power consumption data.

**Figure 17:**
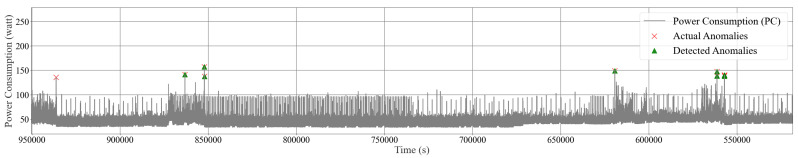
Detected anomalies in the PC power consumption data.

**Figure 18:**
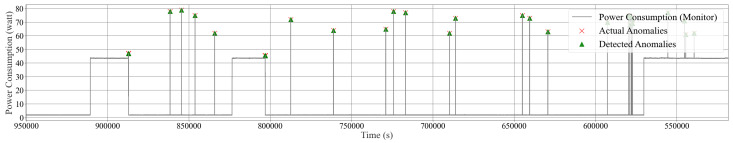
Detected anomalies in the monitor power consumption data.

**Figure 19:**
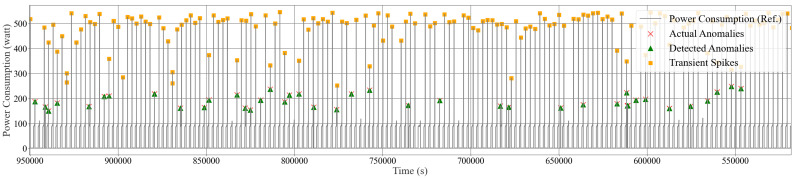
Detected anomalies in the refrigerator power consumption data.

**Figure 20:**
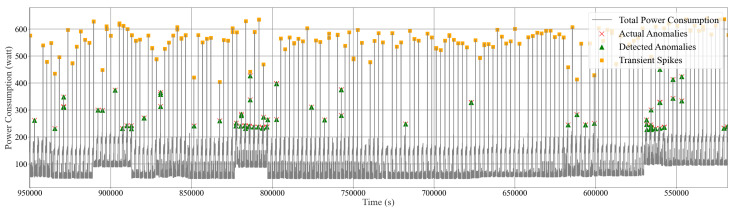
Detected anomalies in overall total power (Home 01) consumption data.

Overall, the averaged performance from the six isolation forest models offers a comprehensive and robust solution for monitoring and maintaining the IIoT system’s integrity, ensuring that any unusual behavior is quickly identified and addressed. Figure 16, Figure 17 and Figure 18 illustrate the anomaly detection for the mini PC, PC, and monitor using the standard isolation forest model. In these figures, the grey dashed line (—) represents the actual power consumption data, the red cross (×) indicates the actual anomalies, and the green triangle (∆) marks the detected anomalies. On the other hand, Figure 19 and Figure 20 illustrate the anomaly detection for the refrigerator and the total power consumption of Home 01 using the optimized isolation forest model while considering transient conditions. In these figures, the grey dashed line (—) represents the actual power consumption data, the red cross (×) indicates the actual anomalies, the green triangle (∆) marks the detected anomalies, and the orange square (□) represents the transient spikes. These models demonstrate promising performance in detecting anomalies, irregular behavior, and transients in real-time. Additionally, this process allows us to identify faulty appliances and detect electricity theft effectively.

### 4.3. Security Verification of the Overall IIoT Infrastructure

**Figure 21:**
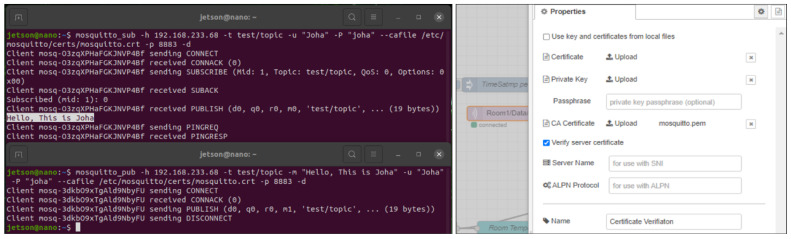
MQTT broker security verification.

The security process begins with implementing TLS/SSL encryption, username and password authentication, and certificate configuration to ensure robust protection for the MQTT broker. This mechanism enhances the security of IIoT applications by preventing unauthorized access and ensuring secure data transmission. This depiction highlights the effective use of secure commands and *cryptographic* measures to ensure protected communication channels within our IIoT environment. The left side of Figure 21 shows the use of a certificate file and a specific user, *Joha*, to securely connect and subscribe to the MQTT topic *test/topic*. The Node-RED configuration panel on the right illustrates the execution of TLS/SSL security using a CA certificate *PEM* file. This setup, shown by the *mosquitto.pem* CA certificate upload option, enables TLS/SSL for secure communication with the broker and ensures server certificate verification, thereby enhancing data transmission security.

For the Node-RED server, it is essential to create TLS/SSL certificates using *OpenSSL* to facilitate HTTPS, thereby securing communications through encryption. We created a certificate, as depicted in Figure S5 (detailed in the Supplementary Materials), issued by and to the entity *Joha* for the organizational unit *Node-red-server*, which highlights its specific use in securing a Node-RED server environment.

### 4.4. Demonstration of Real-World Applications

This section outlines the step-by-step implementation of the overall system and its real-world applications.

#### 4.4.1. Real-Time Monitoring, Controlling, Scheduling, and Protective System

In many research studies on smart energy management systems within an IIoT environment, there is often a lack of focus on real-time control, scheduling, and protective mechanisms. Researchers nowadays primarily concentrate on the monitoring aspects of smart energy management systems, with a strong emphasis on data collection and analysis. However, real-time control, scheduling, and protection are crucial parts  of a smart energy management system, as they enhance system stability and optimize energy efficiency. In our proposed system, we implemented an IIoT interface, depicted in Figure S6 (detailed in the Supplementary Materials), which includes a comprehensive dashboard essential for real-time monitoring and smart energy management in Room 1 (mini PC). It features integrated controls such as appliance ON/OFF switches for the relay module, alert systems for operational anomalies, and scheduling capabilities, which enhance operational efficiency and safety.

**Figure 22:**
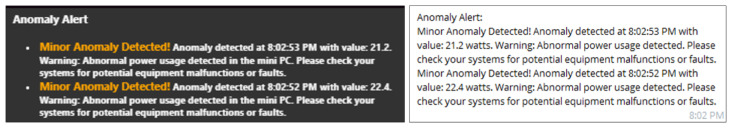
Anomaly detection alert notification.

We applied a condition-based anomaly detection system for power values of anomaly-detected data, categorizing anomalies as minor, moderate, significant, and extreme based on predefined thresholds. The anomaly detection thresholds were set as follows: minor anomalies for power values between 20 and 25, moderate anomalies for values between 25 and 30, significant anomalies for values between 30 and 35, and extreme anomalies for values above 40. This allows for immediate and appropriate responses to varying degrees of electrical irregularities, enhancing system safety and efficiency by alerting operators or users in real-time. When an anomaly is detected, a notification appears on the left side of the Node-RED dashboard, as shown in Figure 22. Simultaneously, a message is instantly sent to the user’s or operator’s smartphone via the Telegram application, displayed on the right side of Figure 22. To enhance the existing anomaly detection logic, an additional condition has been added to automatically turn off the appliance and send a notification to the user when an “extreme anomaly” is detected. Another key feature of our system is that the ESP32 module retains its previous state by storing it in non-volatile memory, such as EEPROM or flash memory. This capability ensures that the module can remember and restore settings, such as relay states, even after a reboot or power cycle. This dashboard is an example of an edge IIoT interface for Room 1. Similarly, we implemented this process for the other rooms and also for the cloud IIoT interface. In the cloud IIoT interface, we deployed the load forecasting AI model instead of the anomaly detection AI model, although deploying the anomaly detection AI model in the cloud is also possible. Since the edge server can quickly collect data and detect anomalies in real-time, we deployed the anomaly detection model on the edge server.

#### 4.4.2. Real-Time Active and Reactive Load Forecasting

In recent years, most studies have focused on active power forecasting, even though reactive power plays a crucial role in power systems. Reactive power is essential for grid stability and energy efficiency, despite sometimes being referred to as wasted power. Understanding and managing reactive power is vital for maintaining the overall reliability and performance of both the electrical grid and the energy distribution system. Our overall system provides insights into one-day-ahead power demand within a secure IIoT environment. This advanced forecasting allows for better planning and scheduling of energy use, leading to optimized energy consumption and cost savings.

**Figure 23:**
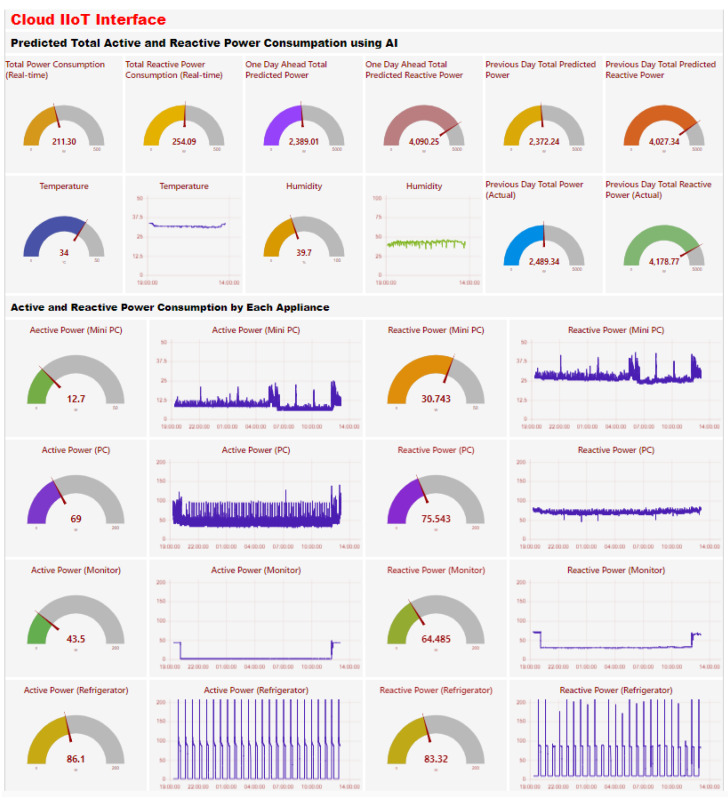
Cloud IIoT layer interface.

The dashboard in Figure 23 displayed the cloud IoT interface, which represents a comprehensive approach to home energy management by using real-time and predictive analytics for active and reactive power consumption. It incorporates environmental data, including temperature and humidity, which are essential for understanding environmental conditions and ensuring human comfort. Furthermore, the detailed breakdown of power usage by individual appliances, such as a mini PC, PC, monitor, and refrigerator, allows users or operators to identify which devices consume the most energy and at what times. This information is critical for implementing targeted energy-saving measures and scheduling appliance usage during off-peak hours to benefit from lower energy rates. The use of visual representations and gauges enhances user engagement and understanding, making it easier to manage energy consumption effectively. Incorporating cloud technology, the system offers scalability and remote accessibility using ZeroTier(1.14.0), ensuring that users can monitor and adjust their energy usage from anywhere. Overall, this smart energy management system illustrates the potential of IoT, AI, and cloud technologies to revolutionize energy management in residential and industrial settings, promoting sustainability and energy efficiency.

The deployment of the TCN-GRU-attention model on the cloud server involved addressing several challenges related to data collection and management, computational complexity, and real-time processing. For efficient data collection, we collected data from sensors in every second, avoiding redundant data storage and unnecessary processing. For data management, we deployed a MariaDB database with SQL queries, integrated with phpMyAdmin for effective handling of large datasets. This allowed for easier retrieval and processing of heterogeneous big data for the AI model. To further enhance the real-time performance of the load forecasting model, we resampled the data from second-level intervals into half-hour periods. This data reduction significantly reducing the number of parameters for the AI model, in turn significantly reducing its computational demands and enhancing real-time processing efficiency. Furthermore, a key challenge in the cloud-based infrastructure was ensuring that the load forecasting script ran automatically and on schedule, eliminating the need for manual execution. To address this, Windows(11) Task Scheduler was configured to trigger the Python(3.10.11) script at designated times each day, allowing for reliable, consistent forecasting. Another key challenge was handling real-time data streams efficiently, especially when dealing with large, complex datasets from multiple sources. To address this, we integrated Node-RED, which effectively manages real-time data, ensuring visualization and continuous monitoring of the AI model’s forecasted data.

#### 4.4.3. Real-Time Anomaly Detection

This procedure helps identify unusual patterns in energy consumption, which can indicate equipment malfunctions, failures, or overloading. Despite its importance, many recent studies fall short of providing effective methods for efficient anomaly detection, highlighting a gap that needs to be addressed in order to improve reliability and performance. Our evaluation focused on assessing the model’s effectiveness in identifying anomalies or unusual behavior in practical applications. We proposed using optimized isolation forest models for anomaly detection, taking into account the transient conditions of appliances like refrigerators, which experience brief electrical spikes during startup or shutdown. These transients are normal and typically managed by the appliances’ internal protection mechanisms, which are designed to handle such fluctuations in electrical current flow. Figure S6 illustrates the real-time anomaly detection, protection, and notification system that we already discussed in the Supplementary Materials. This system is designed to quickly identify anomalies, provide protection against potential issues, and notify users of any unusual behavior. Its real-time capabilities ensure immediate response to anomalies, enhancing the overall efficiency and reliability of operations.

Algorithm 1 outlines a robust approach for real-time anomaly detection (Room 5 of Home 02) in an IIoT environment using MariaDB as the data storage and Pandas for data manipulation. It is designed to fetch sensor data, train an isolation forest model for anomaly detection, and then continuously monitor incoming data for anomalies. The algorithm begins by connecting to the MariaDB database to retrieve historical data, converting these data into a format suitable for analysis (a Pandas dataframe), and reversing the order to ensure it is ordered correctly for analysis. The model training function then takes over, preparing the data by creating an ‘Actual_Anomaly’ column based on a predefined threshold. The data are split into training and testing sets, where the isolation forest model is trained and then saved for future use. After training, the algorithm enters a continuous loop where it fetches the latest sensor data (last single data), applies the pre-trained model to detect anomalies, and inserts these results back into the MariaDB database. This loop runs indefinitely, pausing briefly between each iteration to allow for continuous, real-time monitoring of the IIoT environment. Once the anomalies are detected and inserted into the MariaDB database through the continuous monitoring loop, Node-RED can be configured to pull these data and visualize them on a dashboard.
**Algorithm 1** Anomaly detection and data insertion in MariaDB1:**Input:** table_name2:**Output:** model, metrics3:**function** FETCH_DATA(table_name)4:      Connect to MariaDB and execute SQL query5:      Convert result to DataFrame and reverse order6:      **return** data7:**end function**8:**function** TRAIN_AND_SAVE_MODEL(table_name)9:       df← FETCH_DATA(table_name)10:      **if** df is None or df.empty **then**11:            **raise** Error12:      **end if**13:      $data\leftarrow df[{[}^{\prime }power^{\prime }]]$14:      Prepare data and create ‘Actual_Anomaly’ column15:      Split data into train_data and test_data16:      Train Isolation Forest model and save it17:      Predict anomalies on test_data and calculate metrics18:      **return** model19:**end function**20:**function** INSERT_ANOMALY_DATA_LOOP(model, table_name)21:      **while** True **do**22:            data_new← Latest data from table_name23:            **if** data_new is not None **then**24:                   Predict anomaly and insert result into MariaDB25:            **end if**26:            time.sleep(1)27:      **end while**28:**end function**29:model← TRAIN_AND_SAVE_MODEL(‘room5data’)30:INSERT_ANOMALY_DATA_LOOP(model, ‘room5data’)

Additionally, Node-RED serves as a powerful tool for visualizing data, triggering notifications, and initiating protective measures based on the detected anomalies. This approach ensures that any irregularities in sensor readings are quickly identified and recorded, providing timely insights into potential issues within the industrial or residential system. To implement load forecasting in a cloud server environment, a similar approach to the anomaly detection algorithm is applied. In this scenario, the Python script responsible for load forecasting, which uses a pre-trained model, is scheduled to run at a specific time of the day using the Windows Task Scheduler. This method ensures that the forecasting process is automated and executed reliably according to the defined schedule, making it ideal for regular, timely predictions in a cloud-based infrastructure.

**Figure 24:**
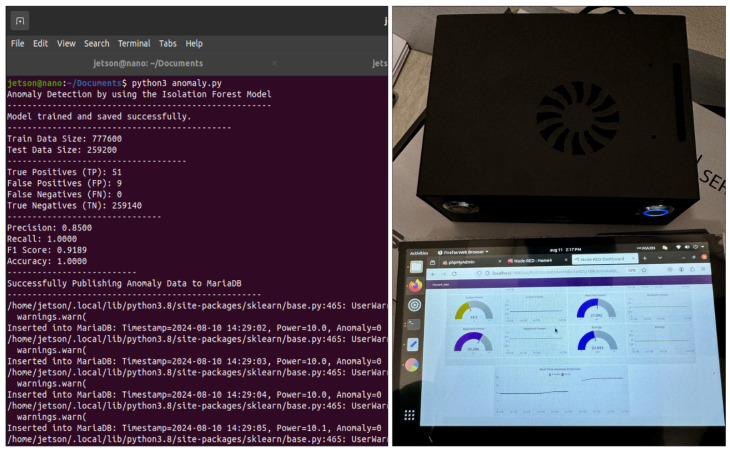
Edge computing on the Jetson Nano board.

Figure 24 illustrates an edge IIoT environment implemented on a Jetson Nano board, featuring an AI model for anomaly detection, MQTT for data communication, a MariaDB database for data storage, and a Node-RED dashboard for real-time visualization. This setup is for Edge Server 02 (Home 02), which monitors and controls an LED light, with the Jetson Nano board implemented as the edge computing device. However, additional appliances can easily be integrated into the system in Home 02. On the left, where the anomaly detection AI model is implemented on the Jetson Nano board, the terminal output indicates the successful training of an isolation forest anomaly detection model, which achieved a precision of 0.8500, recall of 1.0000, F1 score of 0.9189, and perfect accuracy of 1.0000 in identifying anomalies. The model’s results are published in the MariaDB database, with timestamps, actual power values, and detected anomalies. On the right side, the setup displays the Jetson Nano board running a Node-RED dashboard, connected to a screen, and displaying real-time power metrics and anomaly detection results. This setup highlights the Jetson Nano’s ability to manage AI processing, data communication, managing a database, and visualization simultaneously in an industrial or residential edge computing environment. To ensure efficient data transmission, we implemented the MQTT protocol, which significantly reduces communication delays between the sensors and the edge servers. The anomaly detection model operates continuously, as shown in Figure 24, fetching high-frequency sensor data every second to identify potential anomalies, ensuring timely detection and quick system response. Additionally, the system latency from the Python script to the data being sent to the database is approximately 5 milliseconds (0.005 s), ensuring minimal delay in processing and real-time monitoring.

In deploying real-time anomaly detection in the edge servers, we faced several challenges. The first challenge was the need for efficient data handling, as the model fetches data every second from multiple sources and processes it in real-time to check for anomalies. To address this, we implemented local data preprocessing and utilized MQTT for efficient data transmission, which significantly reduced communication delays between the sensors and the edge servers. Another challenge was providing real-time visualization of anomalies for the user. Once anomalies were detected and stored in MariaDB, we configured Node-RED to display this data on a dashboard, enabling users to track anomaly trends instantly and enhancing the system’s usability and reliability. Finally, we faced the challenge of alerting users promptly when anomalies were detected. To solve this, we implemented a notification system where, upon anomaly detection, a message was sent to the user’s smartphone via the Telegram application, in addition to a visual notification on the Node-RED dashboard. This ensures that users are alerted instantly, both visually and via mobile notifications, improving system responsiveness.

## 5. Conclusions

This study introduces a robust and secure IIoT framework that integrates real-time active and reactive load forecasting with advanced anomaly detection, effectively addressing key challenges and limitations found in existing research. It presents the integration of MariaDB and SQL queries within a secure IIoT environment, facilitating the efficient management of heterogeneous big data. By integrating smart data acquisition systems with real-time monitoring, control, and protective measures, the proposed approach ensures secure and reliable industrial operations. The simultaneous forecasting of active and reactive power, combined with advanced anomaly detection, optimizes energy usage and mitigates potential equipment failures, contributing to grid stability and sustainability. The deployment of AI models on both the cloud or centralized server and a single-board computer (SBC) like Jetson Nano demonstrates the practical viability of this system for real-world applications.

We propose a TCN-GRU-attention model for predicting both active and reactive loads, which outperforms traditional models. The performance metrics for active load forecasting include an MSE of 0.0183, MAE of 0.1022, and RMSE of 0.1354. For reactive load forecasting, the model achieves an MSE of 0.0202, MAE of 0.1077, and RMSE of 0.1422. Additionally, we deploy an optimized isolation forest model for anomaly detection that considers the transient states of appliances when detecting abnormal conditions. This model shows excellent results, with average performance metrics across all appliances reaching 95% precision, 98% recall, a 96% F1 score, and nearly 100% accuracy. The system also features alert notifications and protective measures upon detecting anomalies, while visualizing the data on a Node-RED dashboard. To secure the overall system, we implement transport layer security (TLS) and secure sockets layer (SSL) protocols, along with hash-encoded encrypted credentials, to provide robust protection. This research not only addresses key gaps in the existing literature but also provides a practical and scalable solution for modern industrial applications, ultimately contributing to more resilient, sustainable, and secure industrial processes.

Although we achieved promising results in forecasting active and reactive loads, a limitation of this study is that there is still room for improvement in forecasting accuracy. The dataset used includes four months and ten days of data, which limits our ability to account for seasonality in one-day-ahead forecasts. With a full year of data, we could better incorporate seasonal patterns and potentially achieve more accurate forecasting results. Since our developed integrated system is a robust, comprehensive, and practical solution for an IIoT environment with smart energy management, we can expand its application to integrate with smart microgrids, smart grids, and virtual power plants. Furthermore, we could also deploy non-intrusive load monitoring (NILM), federated learning, explainable AI, and blockchain technologies to further enhance the system’s reliability and efficiency.

## Figures and Tables

**Table 1:** Hyperparameters of the proposed TCN-GRU-attention model.

Model	Parameters	Values
TCN-GRU-Attention	Kernel Size	3
	No. of Filters	32
	No. of GRU Units	64
	Learning Rate	0.001–0.00001
	Loss Function	MSE
	Batch Size	64
	Optimizer	Adam
	No. of Training Epochs	200

**Table 2:** Hyperparameters of the proposed isolation forest model.

Appliance	n_estimator	Contamination	Threshold
Mini PC	200	0.0009	20
PC	200	0.001	135
Monitors	200	0.0065	45
Refrigerator	100	0.01	150
LED Light	200	0.006	12
Total Power	100	0.0045	230

**Table 3:** Comparing the proposed model during active load forecasting.

Method	MSE	MAE	RMSE
LSTM	0.0214	0.1123	0.1465
GRU	0.0207	0.1100	0.1440
TCN	0.0187	0.1060	0.1367
Stacked-LSTM	0.0229	0.1136	0.1513
Stacked-GRU	0.0193	0.1064	0.1392
CNN-LSTM	0.0270	0.1213	0.1644
CNN-GRU	0.0229	0.1121	0.1513
LSTM-Attention	0.0215	0.1136	0.1469
GRU-Attention	0.0212	0.1149	0.1457
**TCN-GRU-Attention**	**0.0183**	**0.1022**	**0.1354**

**Table 4:** Comparing the proposed model during reactive load forecasting.

Method	MSE	MAE	RMSE
LSTM	0.0255	0.1224	0.1599
GRU	0.0245	0.1188	0.1565
TCN	0.0225	0.1180	0.1501
Stacked-LSTM	0.0250	0.1184	0.1584
Stacked-GRU	0.0216	0.1140	0.1471
CNN-LSTM	0.0279	0.1229	0.1671
CNN-GRU	0.0257	0.1165	0.1604
LSTM-Attention	0.0231	0.1167	0.1521
GRU-Attention	0.0221	0.1146	0.1487
**TCN-GRU-Attention**	**0.0202**	**0.1077**	**0.1422**

**Table 5:** Performance analysis of the proposed anomaly detection models.

Appliance	Precision	Recall	F1 Score	Accuracy
Mini PC	0.9646	1	0.9820	1
PC	1	0.9298	0.9636	0.9999
Monitors	0.9437	1	0.9711	1
Refrigerator	1	1	1	1
LED Light	0.8500	1	0.9189	1
Total Power	0.9286	0.9778	0.9526	0.9999
**Average Performance Metrics**	**0.95**	**0.98**	**0.96**	**1**

## Data Availability

The data presented in this study are available on request from the corresponding authors.

## Supplementary Materials

The following supporting information can be downloaded at: https://www.mdpi.com/article/10.3390/s24237440/s1, Figure S1: Proposed architecture for the sensing layer; Figure S2: Dilated causal convolution operation; Figure S3: The architecture of an attention layer; Figure S4: Configuration of the sensing layer; Figure S5: Node-RED server security verification; Figure S6: Edge IIoT layer interface; Table S1: Common IoT devices of the IIoT infrastructure; Algorithm S1: Secure communication of an ESP32 module to a WiFi network and an MQTT broker; Algorithm S2: Fetch data from MariaDB and process it using pandas.
