# Supplementary material for: A Secure IIoT Environment That Integrates AI-Driven Real-Time Short-Term Active and Reactive Load Forecasting with Anomaly Detection: A Real-World Application
Source: Sensors (Basel). 2024 Nov 21;24(23):7440. doi: 10.3390/s24237440 (PMC11644684; doi:10.3390/s24237440)
Supplement: Supplementary file 1 [file sensors-24-07440-s001.zip › sensors-3310579-supplementary.pdf]

sensors

Supplementary Materials for: A Secure IIoT Environment That Integrates AI-Driven Real-Time Short-Term Active and Reactive Load Forecasting with Anomaly Detection: A Real-World Application

Md. Ibne Joha, Md Minhazur Rahman, Md Shahriar Nazim and Yeong Min Jang *

Department of Electronics Engineering, Kookmin University, Seoul 02707, Republic of Korea; ibne.joha17@gmail.com (M.I.J.); minhaz.eee.97@gmail.com (M.M.R.); shahriarnazim45@gmail.com (M.S.N.)
* Correspondence: yjang@kookmin.ac.kr; Tel.: +82-2-910-5068

check for updates

Citation: Joha, M.I.; Rahman, M.M.; Nazim, M.S.; Jang, Y.M. *Supplementary Materials for:* A Secure IIoT Environment That Integrates AI-Driven Real-Time Short-Term Active and Reactive Load Forecasting with Anomaly Detection: A Real-World Application. *Sensors* **2024**, *24*.

Received: 28 October 2024
Revised: 18 November 2024
Accepted: 19 November 2024

Copyright: © 2024 by the authors. Licensee MDPI, Basel, Switzerland. This article is an open access article distributed under the terms and conditions of the Creative Commons Attribution (CC BY) license (https://creativecommons.org/licenses/by/4.0/).

1. Introduction

1.1. Literature Review

1.2. Contribution

2. Methodology

2.1. System Overview

2.2. Sensing Layer

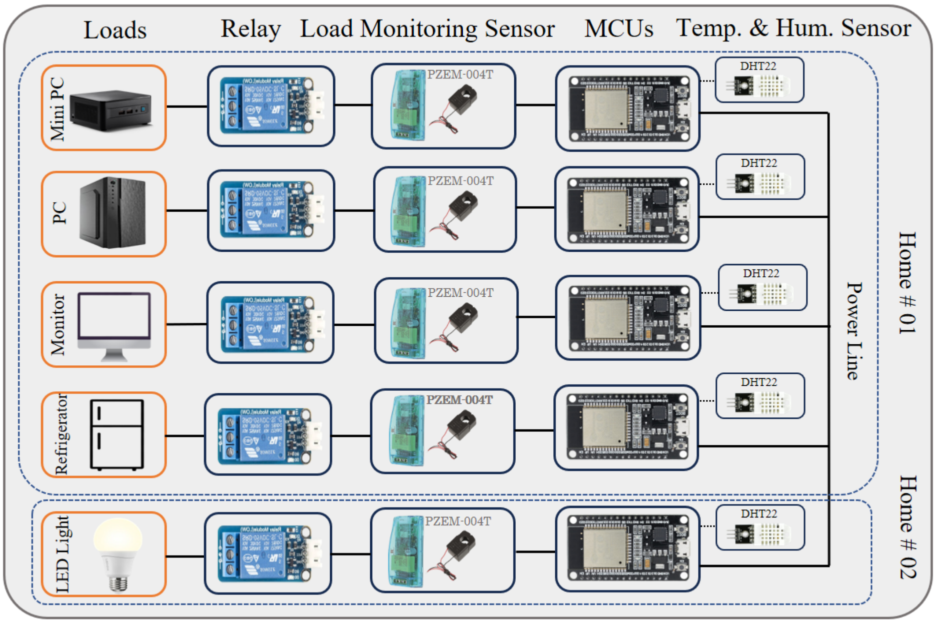

Figure S1. Proposed architecture for the sensing layer.

Figure S1 illustrates a comprehensive energy management system implemented in two distinct homes, designated as Home 01 and Home 02. Home 01 includes various electrical loads such as a mini PC, PC, Monitor, and Refrigerator, while Home 02 features a single electrical load: an LED light. In the modern era, the definition of home appliances has broadened to include electronic devices like mini PCs, PCs, and monitors, which are essential for work, education, and entertainment. Their widespread use and impact on daily life reflect their role as integral components of contemporary households. Although my experiment focuses on using these appliances as electrical loads, we can also incorporate other home appliances without significant issues. These loads are controlled and

monitored via a series of interconnected devices, with each load connected to a relay module responsible for switching the device on and off. Following the relay module, a load monitoring sensor, specifically the PZEM-004T [1], is installed to measure the electrical parameters. The PZEM-004T sensor is capable of measuring various electrical parameters, including voltage, current, power (active power), energy, frequency, and power factor. We can calculate both apparent and reactive power using the following formulas:

Given parameters: V_{rms} (RMS voltage), I_{rms} (RMS current), P (active power in watt). Now, we can use the following formula to calculate apparent power:

$$S = V_{\text{rms}} \times I_{\text{rms}} \tag{S1}$$

Reactive power can be calculated using the Pythagorean theorem:

$$S^2 = P^2 + Q^2 \tag{S2}$$

Solving for Q (Reactive power), we get:

$$Q = \sqrt{S^2 - P^2} \tag{S3}$$

Substituting $S = V_{\text{rms}} \times I_{\text{rms}}$ into the equation, we get:

$$Q = \sqrt{(V_{\text{rms}} \times I_{\text{rms}})^2 - P^2} \tag{S4}$$

Furthermore, we can also calculate apparent and reactive power using the following mathematical equations:
Given parameters: P (active power in watts), $\cos(\theta)$ (power factor).Now, Apparent power can be calculated using the active power and the power factor:

$$S = \frac{P}{\cos(\theta)} \tag{S5}$$

Reactive power can be derived from the relationship between active power, apparent power, and power factor:

$$Q = S \times \sin(\theta) \tag{S6}$$

Where $\sin(\theta)$ can be calculated as:

$$\sin(\theta) = \sqrt{1 - \cos^2(\theta)} \tag{S7}$$

In this study, we used the first measurement process to calculate both reactive power and apparent power. Therefore, the PZEM-004T can measure a wide range of electrical metrics, such as voltage, current, frequency, power factor, active power, reactive power, apparent power, and energy consumption, all of which are crucial for energy management and optimization. The data from the load monitoring sensor is then transmitted to an ESP32 microcontroller, known for its robust performance and versatility in handling multiple sensors and network communications. Each ESP32 microcontroller is also linked to a DHT22 temperature and humidity sensor, enabling the system to monitor environmental conditions in conjunction with electrical load parameters. Each home is equipped with its own set of microcontrollers and sensors, ensuring that energy consumption and environmental data are independently monitored and managed. The structured layout of the system, with distinct pathways for each load type, ensures organized and efficient data management. Overall, sensors gather environmental data and transmit it to the ESP32

module, which then processes the information and publishes it to an MQTT broker under a designated topic.

Algorithm S1 Secure communication of an ESP32 module to a WiFi network and an MQTT broker.

```
Input: ssid, password, mqtt_server, username, pass, ca_cert, sub
Output: Sensor data published to MQTT
function SETUP_WIFI
    Connect to WiFi using ssid and password
end function
function CALLBACK(topic, payload, length)
    if topic matches sub then
        Set relay according to payload
    end if
end function
function RECONNECT
    while not connected to MQTT do
        Attempt to connect to MQTT broker
        if connected then
            Subscribe to necessary topics
        end if
    end while
end function
function SETUP
    Call setup_wifi()
    Set MQTT server and callback function
end function
function LOOP
    if not connected to MQTT then
        Call reconnect()
    end if
    Maintain MQTT connection with client.loop()
    if time since last message > 1000ms then
        Read and publish sensor data
    end if
end function
```

Algorithm S1 is designed to establish a secure connection to a WiFi network and an MQTT broker, read sensor data, and publish this data to an MQTT topic. The setup process initializes the WiFi connection using the provided Service Set Identifier (SSID) and password credentials. It then configures the MQTT server using the specified MQTT server address, username, password, and CA certificate for secure communication. A callback function is set to handle incoming messages on a specific subscription topic *(sub)*. In the main loop, the algorithm continuously checks the MQTT connection and attempts to reconnect if necessary. It maintains the MQTT connection using *client.loop()*. Every second, it reads data from various sensors and publishes this data in JavaScript Object Notation (JSON) format to the MQTT topic. The callback function processes incoming MQTT messages to control the relay state based on the message payload. This design ensures continuous monitoring and remote control of connected devices, facilitating secure data collection and device management over the network.

Table S1 presents the common IoT devices used in this IIoT infrastructure, providing detailed descriptions of each device.

Table S1. Common IoT devices of the IIoT infrastructure.

IoT Devices	Details
Temperature and Humidity sensor (DHT22 or AM2302)	Temperature Range: -40 to 80 °C<br>Temperature Accuracy: ±0.5 °C<br>Humidity Range: 0 to 100% RH<br>Humidity Accuracy: ±2-5% RH
Relay Module	Operating Voltage: 5V DC<br>Rating: 10A at 250V AC<br>Input Current: 20mA at 5V DC (control side)<br>Switching Time: < 10ms (on/off)
Wi-Fi enabled microcontroller (ESP 32 Module)	Processor: Dual-core Tensilica LX6<br>Clock Speed: Up to 240 MHz<br>Flash Memory: 4MB<br>Wi-Fi: 802.11 b/g/n
Energy monitoring sensor (PZEM-004T)	Voltage: 80 to 260V AC, Ac: ±0.5%<br>Current: 0 to 100A, Ac: ±0.5% %<br>Power : 0 to 23kW,Ac: ±0.5% %<br>Frequency: 45 to 65Hz, Ac: ±0.5%<br>Power Factor: 0.00 to 1.00, Ac: ±1%<br>Energy : 0 to 9999kWh, Ac: ±0.5%
Raspberry Pi 4 Model B	Processor: Quad-core Cortex-A72 (1.5 GHz)<br>Wi-Fi: 802.11 b/g/n/ac
Jetson Nano	Processor: NVIDIA Quad-core ARM Cortex-A57 (1.43 GHz)<br>GPU: NVIDIA Maxwell GPU with 128 CUDA cores (921 MHz)<br>Memory: 4 GB LPDDR4<br>OS: Ubuntu 20.04
Mini PC	Processor: Intel (R) N100 (0.8 GHz)<br>GPU: Intel (R) UHD Graphics<br>RAM: 8.0 GB DDR4<br>OS: Windows 11
Centralized Server PC	Processor: 12th Gen Intel (R) Core (TM) i7-12700 (2.10 GHz)<br>GPU: NVIDIA GeForce RTX 3060 Ti<br>RAM: 32.0 GB DDR5<br>OS: Windows 11 Pro

2.3. Edge IIoT Layer

2.4. Centralized IIoT Layer

2.5. Implementation of AI Models

2.5.1. Active and Reactive Load Forecasting

Algorithm S2 Fetch data from MariaDB and process it using pandas.

```
Input: table_name
Output: data
function FETCH_DATA(table_name)
    try
        conn ← mariadb.connect(user, password, host, port, database)
        cursor ← conn.cursor()
        query ← f"SELECT * FROM {table_name} ORDER BY id DESC LIMIT ..."
        cursor.execute(query)
        data ← pd.DataFrame(cursor.fetchall(),
            columns=[desc[0] for desc in cursor.description])
        data ← data.iloc[:: −1].reset_index(drop = True)
        return data
    except mariadb.Error as e:
        print(f"Error connecting to MariaDB Platform: {e}")
        return None
    finally
        if conn then
            conn.close()
end function
room1data ← FETCH_DATA('room1data')
room2data ← FETCH_DATA('room2data')
room3data ← FETCH_DATA('room3data')
room4data ← FETCH_DATA('room4data')
room5data ← FETCH_DATA('room5data')
```

Algorithm S2 effectively demonstrates how to connect to a MariaDB database, retrieve a large dataset, and load it into a Pandas DataFrame for each specified table. This function connects to the database using specific credentials, executes a query to fetch the rows from a given table ordered by id in descending order, and then loads the data into a Pandas DataFrame. The DataFrame is subsequently reversed to maintain ascending order, ensuring data consistency for time series analysis. This approach uses the powerful querying capabilities of SQL and the flexibility of pandas for post-processing. It also includes error handling to manage potential database connection issues, ensuring robustness and reliability. This data fetching process is applicable to both edge and cloud or centralized IIoT layers, ensuring a comprehensive and efficient data acquisition system.

2.5.2. Anomaly Detection

2.6. Model Description

2.6.1. Load Forecasting Model

Figure S2 demonstrates a dilated causal convolution operation, essential for handling sequential data. It ensures that outputs at each time step rely on current and past inputs, preserving temporal integrity. The model uses increasing dilation factors across layers, allowing it to capture broader temporal patterns efficiently.

Figure S3 highlights the importance of the attention mechanism in sequential modeling, as it enables the model to concentrate on the most relevant portions of the input sequence.

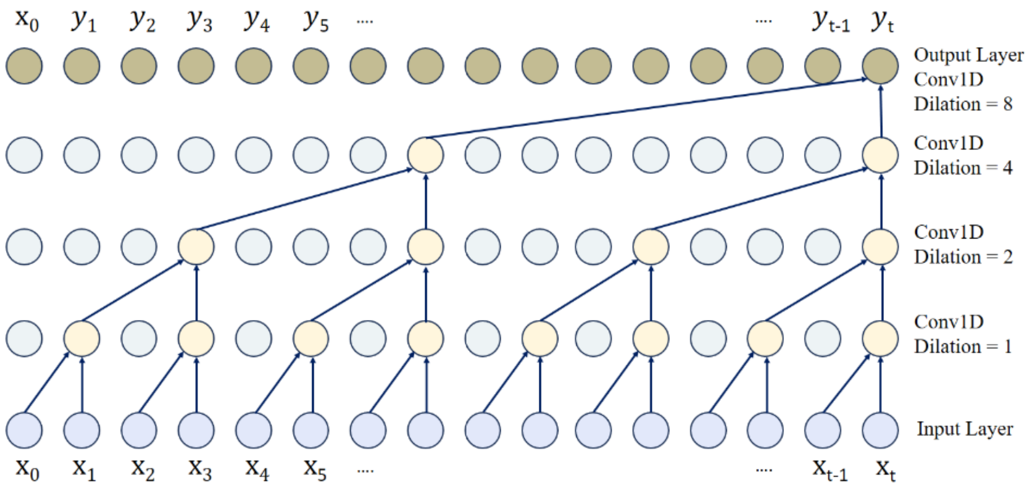

Figure S2. Dilated causal convolution operation.

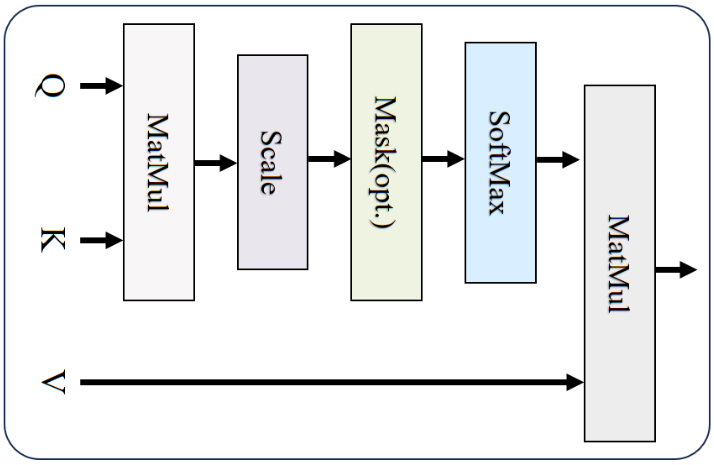

Figure S3. The architecture of an attention layer.

2.6.2. Anomaly Detection Model

2.6.3. Evaluation Metrics

2.7. Securing The Overall IIoT Infrastructure

3. Experimental Setup

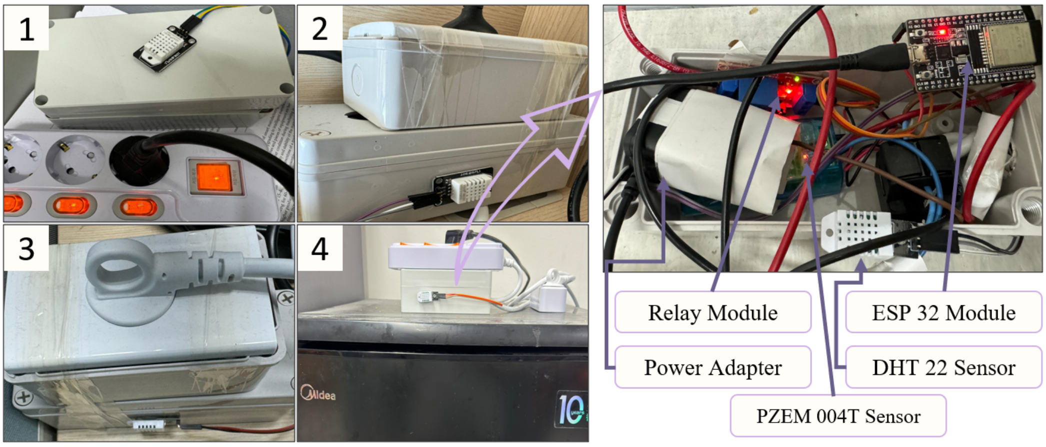

Figure S4. Configuration of the sensing layer.

3.1. Experimental Setup of Sensing Layer

The smart data acquisition system in Figure S4 features a rigid and compact setup, which is both portable and secure. By enclosing all components within a sealed plastic case, it ensures maximum safety, preventing exposure to electrical parts and mitigating any risk of accidental contact or damage. The right side of the figure provides a detailed view of the smart data acquisition system (SDAS) internal components, including the ESP32 module, relay module, power adapter, DHT22 sensor, and PZEM 004T sensor. This setup highlights the device's capability to manage, monitor, and control various electrical parameters and appliances within an IIoT system, ensuring efficient and safe operation. The application of the smart data acquisition system (SDAS) is connected to various appliances: a mini PC (Room 1), a PC (Room 2), monitors (Room 3), and a refrigerator (Room 4), each located in different rooms. Additionally, an LED light equipped with SDAS is installed in a separate room, identified as Home 2 (Room 5). These appliances are controlled by a relay, enabling remote control and protective functionality, which enhances safety and efficiency within the IIoT framework.

3.2. Experimental Setup of Edge IIoT Layer

3.3. Experimental Setup of Centralized IIoT Layer

4. Experimental Results

4.1. Dataset Description

4.2. Performance Evaluation of AI Models

4.2.1. Active and Reactive Load Forecasting

4.2.2. Anomaly Detection

4.3. Security Verification of the Overall IIoT Infrastructure

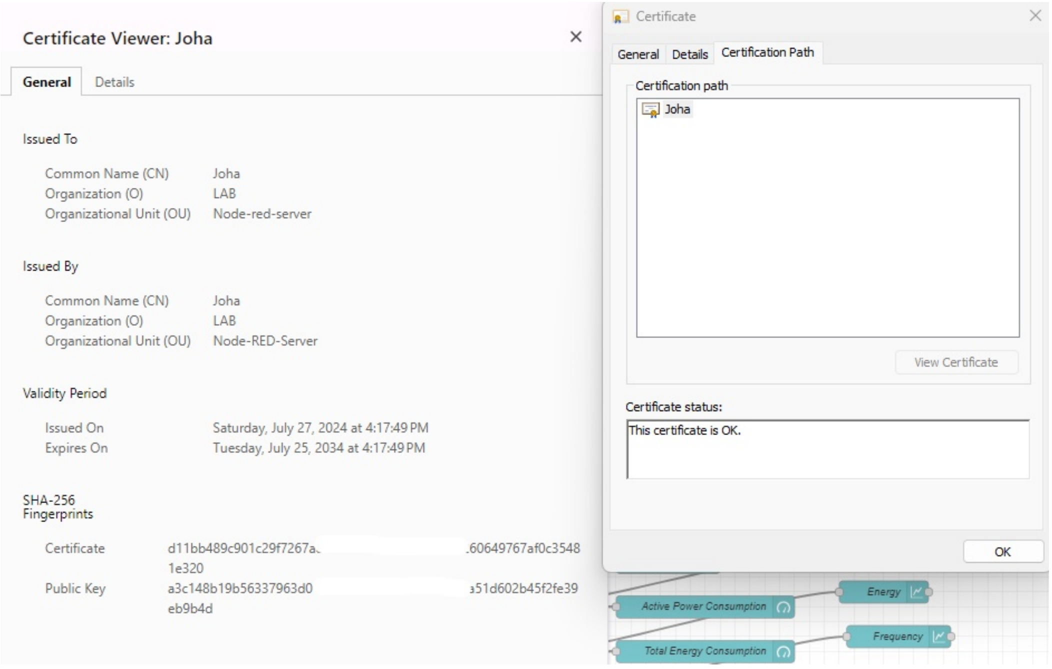

Figure S5. Node-RED server security verification.

For the Node-RED server, it is essential to create TLS/SSL certificates using *OpenSSL* to facilitate HTTPS, thereby securing communications through encryption. We created a certificate, as depicted in Figure S5, issued by and to the entity *Joha* for the organizational unit *Node-red-server* which highlights its specific use in securing a Node-RED server environment. It is valid from July 27, 2024, to July 25, 2034, highlighting a ten-year validity period that ensures sustained encryption protection for the server. The certificate status is confirmed as *OK*, ensuring that it is valid and properly configured for secure operations

within the designated system. Additionally, the certification path reveals that the certificate is *self-signed*, being issued by the same entity it certifies, which serves to establish a trusted communication channel. Furthermore, on the Node-RED server, we implemented a hashed password generation process that facilitates the use of a username and hashed password for authentication. This method ensures that access is strictly limited to authorized users, thereby enhancing the server's security with robust authentication credentials. We used the *mysql_secure_installation* script to enhance the security of our MariaDB setup by setting a strong root password, removing anonymous users, and limiting remote root access. For phpMyAdmin, we configured Apache and used a dedicated user with strong credentials instead of the root account. Finally, we used ZeroTier for secure remote access, which offers end-to-end encryption and an isolated virtual network.

4.4. Demonstration of Real-World Applications

4.4.1. Real-Time Monitoring, controlling, Scheduling, and Protective System

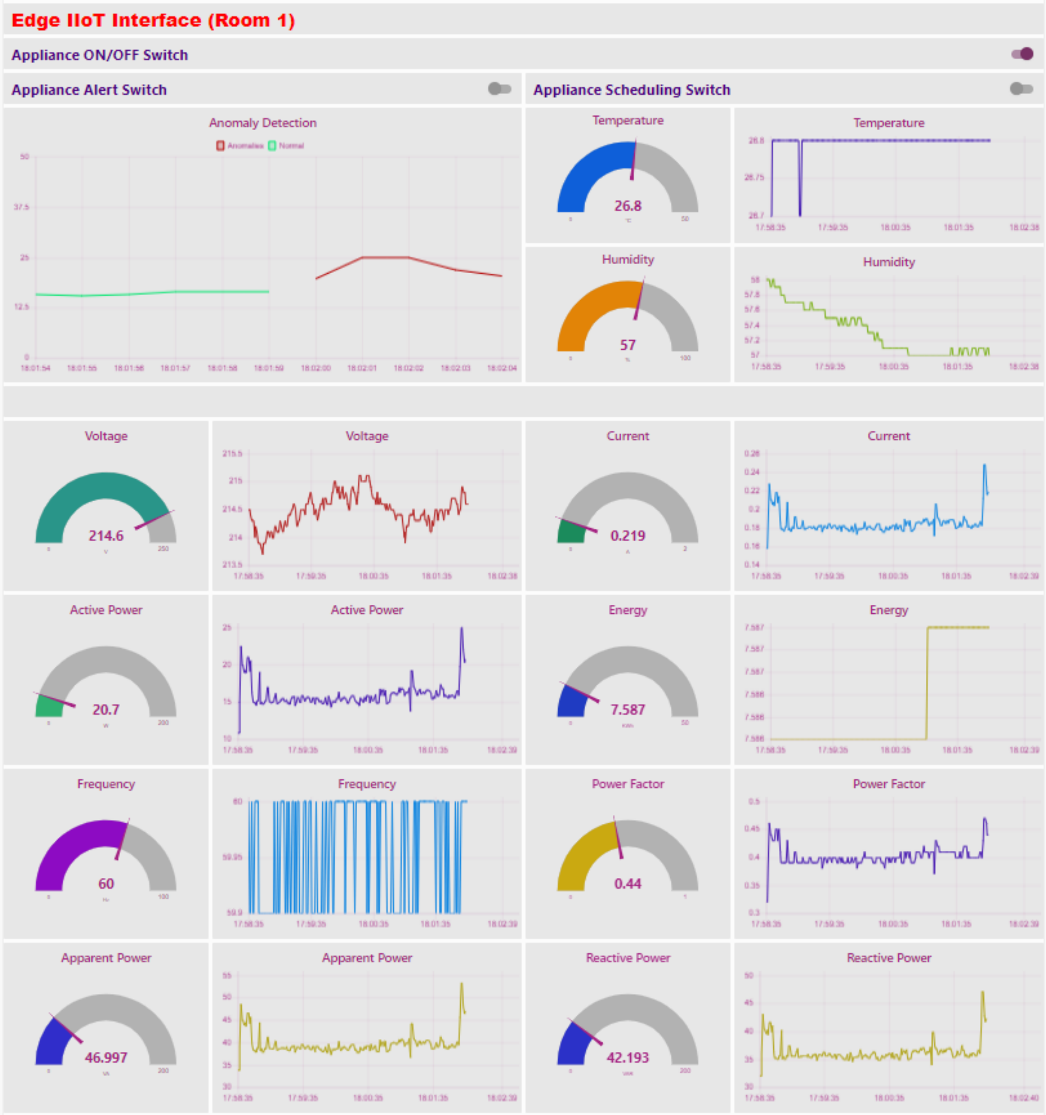

Figure S6. Edge IIoT layer interface.

In many research studies on smart energy management systems within an Industrial Internet of Things (IIoT) environment, there is often a lack of focus on real-time control, scheduling, and protective mechanisms. Researchers nowadays primarily concentrate on the monitoring aspects of smart energy management systems, with a strong emphasis on

data collection and analysis. However, real-time control, scheduling, and protection are crucial parts of a smart energy management system, as they enhance system stability and optimize energy efficiency. In our proposed system, we implemented an IIoT interface, depicted in Figure S6, which includes a comprehensive dashboard essential for real-time monitoring and smart energy management in Room 1 (Mini PC). It features integrated controls such as appliance ON/OFF switches for the relay module, alert systems for operational anomalies, and scheduling capabilities, which enhance operational efficiency and safety. Users or operators can schedule their appliances to turn on or off as needed. By providing real-time data on parameters such as voltage, current, active power, energy usage, frequency, power factor, apparent power, and reactive power the dashboard allows for precise monitoring and control over electrical systems, minimizing the risk of overload and optimizing power consumption. Environmental metrics like temperature and humidity ensure conditions stay within safe and efficient limits, crucial for both sensitive industrial processes and human comfort. Additionally, the Anomaly Detection graph plays a vital role by alerting operators to deviations in normal operational parameters, potentially preventing equipment failures or overloading. It uses red to signal anomalies and green to indicate normal conditions, facilitating the quick identification of irregular system behavior. Moreover, the ability to schedule appliances and control them remotely not only optimizes energy usage but also extends the lifespan of the equipment by preventing overuse and ensuring efficient energy management.

4.4.2. Real-Time Active and Reactive Load Forecasting

4.4.3. Real-Time Anomaly Detection

5. Conclusion

References

1. Sadeeq, M.A.; Zeebaree, S.R. Design and implementation of an energy management system based on distributed IoT. *Comput. Electr. Eng.* **2023**, *109*, 108775.

Disclaimer/Publisher's Note: The statements, opinions and data contained in all publications are solely those of the individual author(s) and contributor(s) and not of MDPI and/or the editor(s). MDPI and/or the editor(s) disclaim responsibility for any injury to people or property resulting from any ideas, methods, instructions or products referred to in the content.
